# The effectiveness of Ni@SiTiCNO nanocomposite coating for protecting steel used in agricultural machinery dealing with animal waste

**DOI:** 10.1038/s41598-026-47435-4

**Published:** 2026-04-17

**Authors:** Gamal E. M. Nasr, Mohamed A. Refai, Aliaa G. Abd Elaziz, Mona H. Gomaa, Said M. El-Sheikh, Yasser M. Z. Ahmed, Z. Abdel Hamid

**Affiliations:** 1 Agricultural Engineering Department, Fac. Agric., Cairo University, Giza, 12613 Egypt; 2 Corrosion Control and Surface Protection Department, Metals Technology Institute, (CMRDI), Cairo, Egypt; 3 Department of Ceramic and Refractory, Central Metallurgical R&D Institute, Cairo, 11421 Egypt; 4 Department of Nanomaterials and Nanotechnology, Central Metallurgical R&D Institute, Cairo, 11421 Egypt

**Keywords:** Agricultural machinery, Nanoparticles, Nanocomposite, Electrodeposition, Corrosion resistance, Chemistry, Engineering, Environmental sciences, Materials science, Nanoscience and technology

## Abstract

Because corrosive environments are created, agricultural equipment used in livestock farms and poultry houses that handle animal waste is vulnerable to corrosion. Therefore, the purpose of this work was to increase the corrosion resistance of AISI 1020 low-carbon steel by applying protective Ni@SiTiCNO nanocomposite coatings using the electrodeposition technique, where ‘@’ denotes the incorporation of nanoparticles into the matrix, not a core-shell structure. Fourier Transform Infrared Spectroscopy (FT-IR), Raman, Field Emission Scanning Electron Microscopy (FE-SEM, QUANTA EG) with an energy dispersive X-ray system (FE-SEM, EDS), and High-Resolution Transmission Electron Microscopy (HR-TEM) have all been used to study the structure and morphology of the SiTiCNO nanoparticle. Additionally, the surface area and optical characteristics were examined. During the deposition process, various SiTiCNO nanoparticle concentrations (0–2 g/L) and current densities (3–5 A/dm^2^) were employed. The surface morphology, corrosion resistance (in urea 3.5%), and abrasion behavior have all been thoroughly examined. According to the corrosion resistance results, uncoated steel had a high corrosion rate (0.556 mm/a), whereas Ni@SiTiCNO deposited at 5/Adm^2^ from the Watts electrolyte bath containing 2 g/L SiTiCNO nanoparticle had the lower corrosion rate (0.008 mm/a) means the highest corrosion resistance.

**Supplementary Information:**

The online version contains supplementary material available at 10.1038/s41598-026-47435-4.

## Introduction

Farm mechanization plays the main role in achieving the sustainability goals of the United Nations. This is because it significantly contributes to increasing production efficiency, enhancing product quality, and expediting operational processes, which can also help rural communities financially due to increased production^1,2^.

Agricultural machinery wear and corrosion are problems, and new studies have shown how urgently better materials and preventive techniques are needed to lower these financial losses^3,4^. At modern animal farms, animal waste management equipment is extremely vulnerable to rapid metal corrosion because of the severe microclimate. The hostile circumstances caused by water vapor, ammonia from animal waste, and high humidity (about 70–72%) in poultry habitats cause chemical and electrochemical reactions on metal surfaces^5^. Through corrosion catalysis and the dissolution of protective oxide layers, these variables result in a large annual loss of metals, up to 460 g/m^2^ in chicken. Ammonia functions as a potent catalyst, and metals are further broken down by organic acids. Therefore, it is crucial to implement cutting-edge corrosion-resistant materials and protective coatings using different techniques such as electrodeposition^6^ and electroless^7,8^.

Moreover, creative technical solutions are needed to prolong the life of equipment and lower maintenance expenses in modern agricultural operations^9^. Nanomaterials have become pivotal tools in advancing composite coatings, delivering substantial improvements in mechanical, chemical, and functional properties. Their nanoscale dimensions impart distinct physicochemical properties that differ fundamentally from their bulk counterparts, thereby enabling exceptional performance in coating applications. Steel’s resistance to corrosion is considerably increased by composite coatings. Because the nanocomposite coating method improves steel’s mechanical, physical, and chemical characteristics, it has turned into a popular surface-engineering trend^10^. In previous studies there are many different nickel nanomaterial composite coatings used to improve wear and corrosion of farm equipment, such as Ni-ZnO^11^, Ni-CNTs^12^, and Ni- TiO_2_^13^ and in other studies there has been a focus on pure Ni coatings for machinery parts dealing with chemical fertilizers^14^ and Ni–Co–SiC^15^.

The recent research confirms that electrodeposited nickel coatings reinforced with ceramic nanoparticles such as zirconia (ZrO_2_), alumina (Al_2_O_3_), and chromia (Cr_2_O_3_) exhibit superior improvements in hardness, wear resistance, and corrosion performance. For instance, Ni–W coatings with ZrO₂ nanoparticles demonstrated microhardness values up to 1074 HV and anticorrosive properties, attributed to the combined effects of grain refinement and nanoparticle dispersion^16^. Furthermore, advanced formulations such as Ni-W/Cr₂O₃ nanocomposites enhance wear resistance by an additional 30%, leveraging synergistic hardening mechanisms. By substantially extending equipment operational lifespan and reducing maintenance frequency, such nanocomposite coatings present a cost-effective strategy for enhancing operational efficiency in modern livestock facilities^17,18^. Silicon carbide (SiC) exhibits exceptional hardness, alongside outstanding wear, oxidation, and corrosion resistance. Promising properties can be attributed to the tetrahedral coordination and the strong covalent bonding of silicon and carbon atoms. These properties can achieve a strong adhesion and high density, which are important parameters in the coating process^19^. By increasing the amount of SiC nanoparticles at the expense of other constituents, the friction coefficient, tends to decrease, which leads to improved transfer layer based on a decrease in the wear rate^20^.

The novel multiphase ceramic nanocomposite SiTiCNO, composed of TiO_2_, TiC, TiC_0.3_N_0.7_, carbon, and SiCN phases, has been synthesized via pyrolysis of a preceramic polymer mixture of polyvinylsilazane and tetrabutyl orthotitanate under a nitrogen atmosphere at temperatures ranging from 900 to 1400 °C. This process results in an amorphous SiCN matrix embedding homogeneously distributed nanocrystals of TiO_2_, TiC, which enhance the composite’s thermal stability and mechanical properties at elevated temperatures. The addition of titanium promotes the formation and ordering of graphitic carbon within the matrix, improving structural integrity. Such polymer-derived ceramic composites have been extensively studied for their high-temperature performance and mechanical robustness, with similar Si-Ti-C-O systems demonstrating excellent resistance to thermal degradation and mechanical wear. Advanced sintering techniques and micro-nano composite designs, including Ti (C, N)-based materials, further illustrate the potential of these multiphase ceramics for demanding structural applications. These findings position SiTiCNO as a promising candidate for high-temperature structural and functional ceramic materials in aerospace, energy, and protective coating industries^21^. On the other hand, carbon nitride Si_3_N_4_ has a low-friction coating in hard disc drives. In addition, it has a high elasticity, good coating adhesion, and adequate hardness, which can reduce wear and subsequently increase the lifetime^22^.

The goal of this work is increasing the low-carbon steel components used in cow dung handling equipment’s resistance to corrosion in the urea environment. SiTiCNO nanocomposite powder synthesized via Sol–Gel technique. The produced nanoparticles’ have been examined using various characterizations. Nanocomposite-coated steel Ni@SiTiCNO; where ‘@’ denotes the incorporation of nanoparticles into the matrix, not a core-shell structure using the electrodeposition process was applied in order to improve the surface characteristics. The impact of various operational parameters, including direct current density and nanoparticle composition, on the physical and chemical characteristics.

of steel coated with composite layers has been investigated. The physical and chemical properties of steel coated with composite layers have been studied in relation to a number of operational parameters, such as direct current density and nanoparticle composition. The investigation’s primary focus was on the shape of the deposited coating and its mechanical properties, such as its resistance to abrasion. Corrosion testing in a 3.5% urea solution was also used to assess the coating’s chemical qualities.

## Materials and methods

This study combines a combination of sol-gel and electrochemical synthesis to install a protective Ni@SiTiCNO hybrid nanocomposite coating on low-carbon steel used in cow manure handling equipment. Firstly, preparation of SiC-Si_3_N_4_-TiC-TiO_2_/SiO_2_ (SiTiCNO) nanocomposite material; the second step is a comprehensive assessment of the nanocomposite through structural characterization (SEM for surface morphology, TEM for nanostructure), chemical analysis (EDX, FTIR, Raman). The third step is preparing the low-carbon steel surface by polishing and cleaning, and finally, the coating is electrodeposited onto steel using a nickel Watt’s bath under direct current (DC) and reinforcement concentrations.

### Preparation of SiC-Si_3_N_4_-TiC-TiO_2_/SiO_2_ (SiTiCNO) nanocomposite materials

 The sol-gel and carbothermal reduction methods have been used to prepare (SiTiCNO) nanocomposites. Table S1 presents the synthesis conditions for the SiC-Si₃N₄-TiC-TiO₂/SiO₂ (SiTiCNO) nanocomposite. The detailed processes are explained in the supplementary file.

### Chemical composition and preparing the steel surface

The chemical composition of steel used in animal waste transport trailers was analysis using PMI-Master smart optical emission spectrometry (OES) analyzer UV Toucher HITACHI Device. According to the manufacturing material’s chemical composition, which is listed in Table 1, it is made of low-carbon steel.

**Table 1:** Chemical composition of low carbon steel.

Elements	C	Mn	Si	*P*	S	Fe
Wt. %	0.15	0.58	0.27	0.02	0.01	Balance.

Using emery sheets with grit sizes ranging from 220 to 1200, the low-carbon steel base metal, which measured 100 mm in length, 20 mm in width, and 0.6 mm in thickness, was polished. This operation was carried out in order to create a bright and clean surface prior to the deposition process starting. The substrate was then degreased in an alkaline solution including trisodium phosphate (Na₃PO₄), sodium hydroxide (NaOH), and sodium carbonate (Na₂CO₃). Deionized water was then used to rinse the substrate. To get rid of any oxide coating, the substrate was then pickled in a solution of 10% hydrochloric acid (HCl). Before being submerged in the plating solution, it was rinsed one more time with deionized water.

###  Procedure for electrodeposition

The manufactured substrate was submerged in the electrolyte solution to act as the cathode (negative electrode) and a nickel plate as the anode (positive electrode) during the electrodeposition procedure. The deposition tests were powered by a Programmable DC Power Supply model TH6600P Series. Electrodeposition was carried out in a 1000 milliliter glass beaker filled with nickel Watts’ bath. Table 2 lists the exact components of the plating solution as well as the particular operating conditions that were applied.

**Table 2:** The chemical composition bath and the operating conditions for preparing Ni@SiTiCNO nano-composite coatings.

Chemical salt	Content(g/L)
NiSO_4_.6H_2_O	250
NiCl_2_.6H_2_O	60
H_3_BO_3_	30
Ni-SiTiCNO	0–2
Operation conditions	
Current density, A/dm^2^	3–5
PH	5
Time (min)	15
Stirring rate (rpm)	400
Temperature ℃	45

Before electrodeposition, SiTiCNO nanoparticles were dispersed in the plating bath at four different concentrations: 0.5, 1.0, 1.5, and 2.0 g/L The suspension was magnetically stirred at 400 rpm for 60 min, then ultrasonicated for 30 min to minimize particle agglomeration. Subsequently, coatings were produced as either pure nickel or Ni@SiTiCNO nanocomposites and examined by the full suite of characterization techniques.

A comprehensive suite of techniques was applied to characterize the Ni@SiTiCNO coating system. Transmission electron microscopy (FEI Tecnai G2 20 S-Twin, 200 kV) determined the primary particle size of the synthesized SiTiCNO nanoparticles. An eddy-current thickness gauge (ElektroPhysik MiniTest 70) was used to measure the average film thickness from multiple measurements. X-ray diffraction (X’Pert PRO—PANalytical, Netherlands) was carried out by employing Cu Kα radiations, λ = 1.5418 Å; a generator voltage of 45 kV; a generator current of 30 mA; and the diffraction angle range (2θ) = 4–80°, step time of 0.4 s, and scan rate of 0.05° s^−1^, yielding crystallographic information, enabling identification of the SiTiCNO phase and estimation of crystallite size. Surface morphology and elemental composition were examined with a field-emission SEM equipped with EDX (QUANTA EG). An IviumStat instrument (provided by Ivium Technologies, Eindhoven, the Netherlands) was used to conduct electrochemical measurements for the corrosion investigation. A 3.5% urea solution made with triply distilled water served as the electrolyte. The investigated electrode was immersed for 60 min. in urea solution until the open-circuit potential (OCP) was recorded, potentiodynamic scans were swept from − 500 mV to + 500 mV versus OCP at 0.1 mV/s. Using coated or bare steel as the working electrode and a surface area of the working electrode 1.0 cm^2^, Pt foil as the counter, and a SCE (saturated calomel electrode), EIS (electrochemical impedance spectroscopy) was obtained between 35 kHz and 100 mHz with a 10 mV AC perturbation.

Finally, an IKA RW 20 digital rotator was used to evaluate abrasion resistance of the investigated samples with dimensions of 20 × 50 × 1 mm tangentially rotated in a water/sand (ratio 3:2) medium. The particle size of the sand ranged from 75 up to 100 μm. The abrasion effect was calculated in terms of weight loss expressed as mg/sliding distance at a constant rotation speed of 300 rpm. The abrasion measurements should be taken within the coating’s thickness without coming into contact with the base metal.

## Results and discussions

### Properties of SiTiCNO Nanocomposite

#### XRD examination

**Fig. 1:**
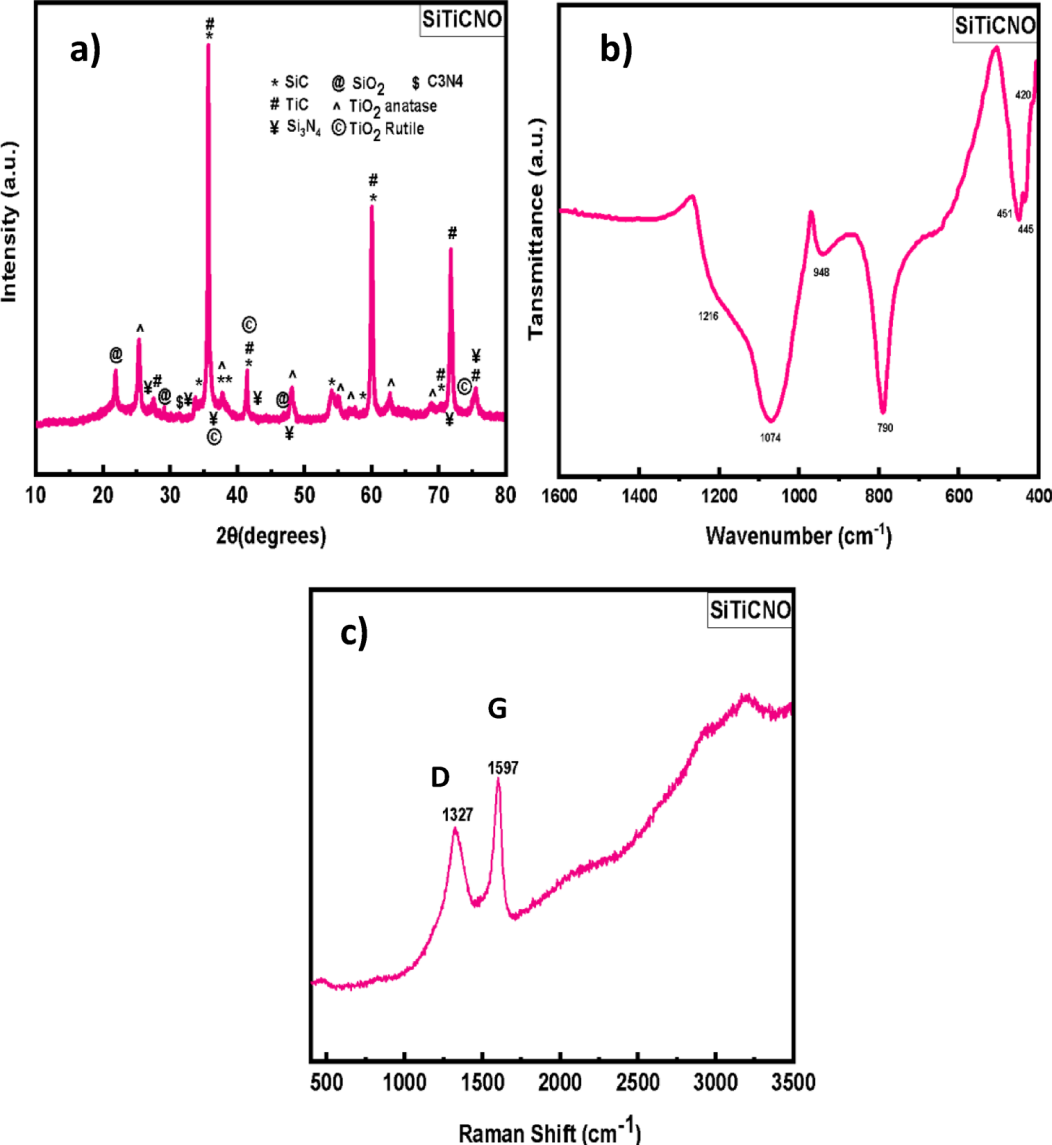
(**a**) XRD, (**b**) FT-IR spectrum, and (**c**) Raman spectroscopy of the synthesized SiTiCNO powder.

Figure 1a and Fig. S1 presents the X-ray diffraction (XRD) patterns of the powder synthesized via carbothermic reduction of a binary xerogel containing silicon and titanium. The main diffraction peaks are attributed to SiC, β-Si₃N₄, and TiC phases^23^. The diffraction peaks observed at 2θ = 33.7°, 34.4°, 35.3°, 35.7°, 37.9°, 38.5°, 41.5°, 54.1°, and 60.0° in line with the (111), (200), (220), and (311) planes of β-SiC, (JCPDS 00–033-1160) in agreement with Liu^24^. Peaks at 2θ = 35.7°, 41.5°, 55.0°, 60.2°, 72.0°, and 75.7° are indexed to TiC (JCPDS card No. 06–0614). A notable peak at 2θ = 33.7° is attributed to stacking faults within the β-SiC structure, as reported in the literature. Additional peaks at 2θ = 33.6°, 36.0°, and 41.5° correspond to β-Si₃N₄^24^ Minor oxide phases, including anatase TiO₂ 2θ = 25.2°, 37.8°, 48.0° (JCPDS No. 01–048-1286)^25^, rutile TiO₂ (2θ = 27.5°, 36.0°, 40.2°, 54.3°; (JCPDS No. 03–035-1119), and SiO₂ 2θ = 21.9° (JCPDS No. 01–082-1403), are also identified. A trace amount of C₃N₄ is indicated by a weak peak at 2θ = 29.1° (01–078-1747)^26^.

#### FTIR spectroscopy

Figure 1b displays the synthesized sample’s FT-IR pattern. A broad, intense band centered at 1074 cm^−1^, spanning the range of 980–1250 cm^−1^, is primarily attributed to Si–C stretching vibrations^24^. Bands in the 1000–1100 cm^−1^ region are associated with Si–N stretching in β-Si_3_N_4_^27^. Furthermore, the asymmetrical stretching resonance of Si–O–Si bonds is associated with the peak at 1040 cm^−1^, whereas Ti–O–Ti stretching in TiO₂ is associated with the peak at 1100 cm^−1^. A distinct peak at 950 cm^−1^ is ascribed to the longitudinal optical (LO) mode of Si–C^28^. Figure 1b further highlights a sharp absorption band centered at 790 cm^−1^, with contributions from both Si₃N₄ (836 cm^−1^, Si–N bonds) and the transverse optical (TO) mode of Si–C. A shoulder at 654 cm^−1^ is also associated with SiC. The Ti–C stretching vibration is observed at 456 cm^−1^^24^. Additional bands in the 434–500 cm^−1^ range are consistent with Si₃N₄ and SiO₂, the latter due to Si–O–Si bending modes, indicative of residual silica following carbothermal reduction^29,30^.

####  Raman spectroscopy

Raman spectroscopy, as shown in Fig. 1c, offers insights into the microstructural features of the carbon phases. Gaussian deconvolution identifies three distinct peaks. The D-band, centered at 1327 cm^−1^, is associated with the breathing modes of disordered carbon rings and indicates sp³-hybridized carbon within SiC–Si₃N₄ matrices^31^. The G-band, centered at 1597 cm^−1^, corresponds to the in-plane stretching of sp^2^ carbon atoms and indicates the development of nanocrystalline graphite^32^. An increased intensity and sharpness of both D and G bands, along with the appearance of the 2D band, suggests enhanced crystallinity of the free carbon. The ID/IG intensity ratio further confirms a reduction in graphite particle size.

#### FE-SEM, EDX, and elemental mapping

**Fig. 2:**
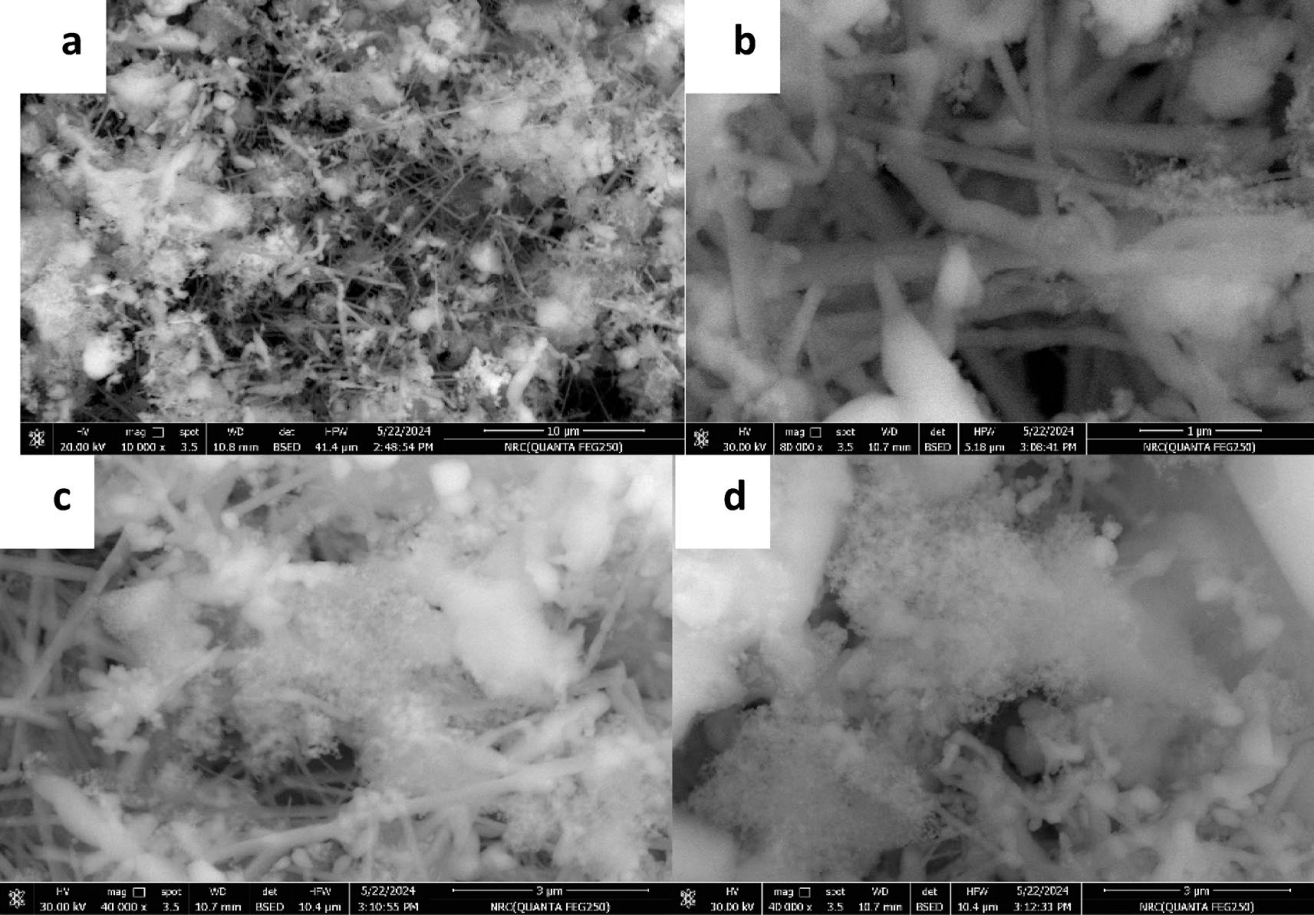
Field-emission scanning electron microscopy (FE-SEM) micrographs, presented in (**a**) the general morphology, (**b**) highlights elongated SiC extending several microns in length, (**c** and **d**) darker spherical nanoparticles.

FE-SEM micrographs (Fig. 2a–d) reveal the morphological features of the synthesized sample. Figure 2a illustrates the general morphology, while Fig. 2b highlights elongated SiC nanorods, extending several microns in length. Figure 2c and d show darker spherical nanoparticles, potentially corresponding to nano-β-Si₃N₄, along with irregular, block-like features attributed to TiC. Spherical SiO₂ particles and ultrafine cotton-like TiO₂ nanoparticles are also visible^24^.

**Fig. 3:**
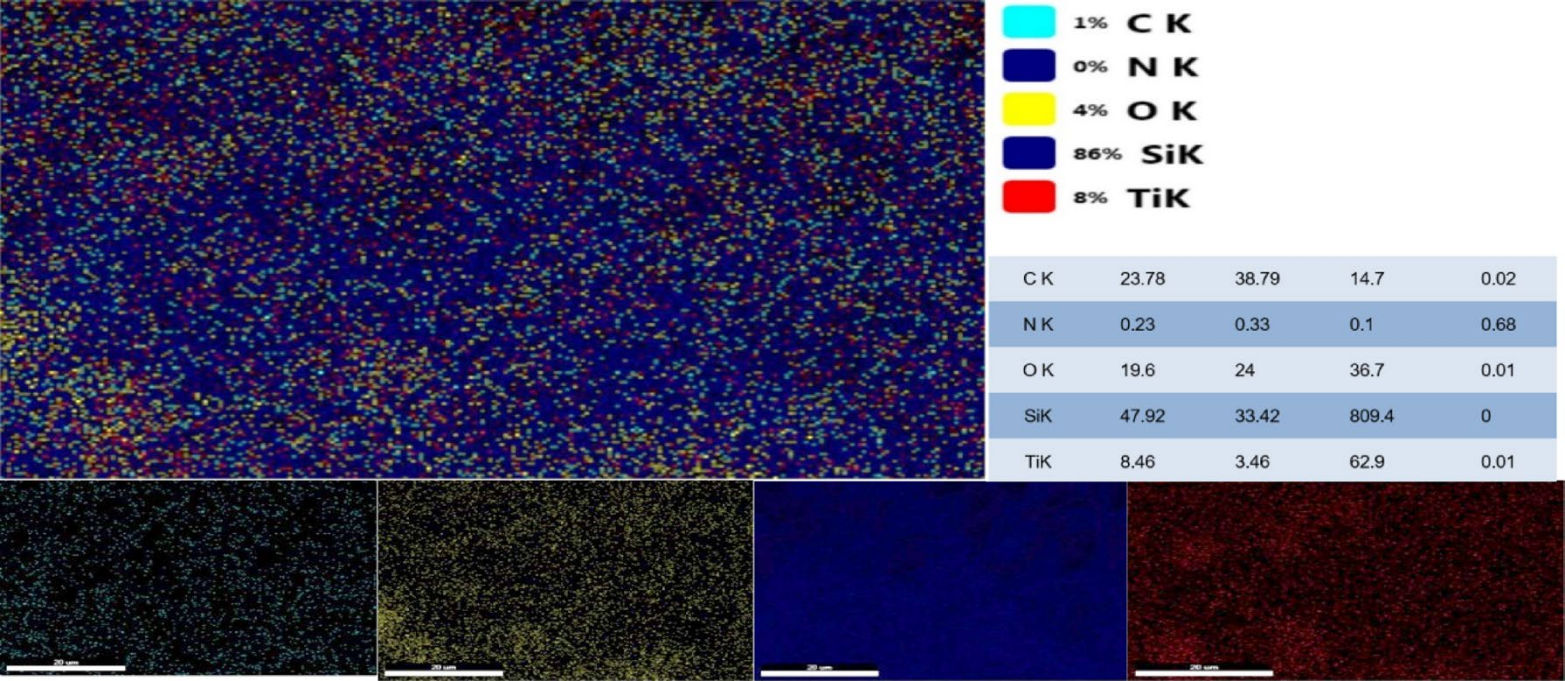
Demonstrates uniform distribution of elements (mapping), corroborating EDX results.

Elemental analysis via EDX confirms the presence of C, O, N, Si, and Ti. High carbon and silicon concentrations support the predominance of SiC, with minor TiC phases^33^. The relatively low nitrogen content suggests limited β-Si₃N₄ formation. Oxygen presence indicates the formation of TiO₂ and SiO₂ phases^26^. Elemental mapping (Fig. 3) demonstrates uniform distribution of elements, corroborating EDX results and confirming the homogeneity of the sample^27^.

#### TEM analysis

**Fig. 4:**
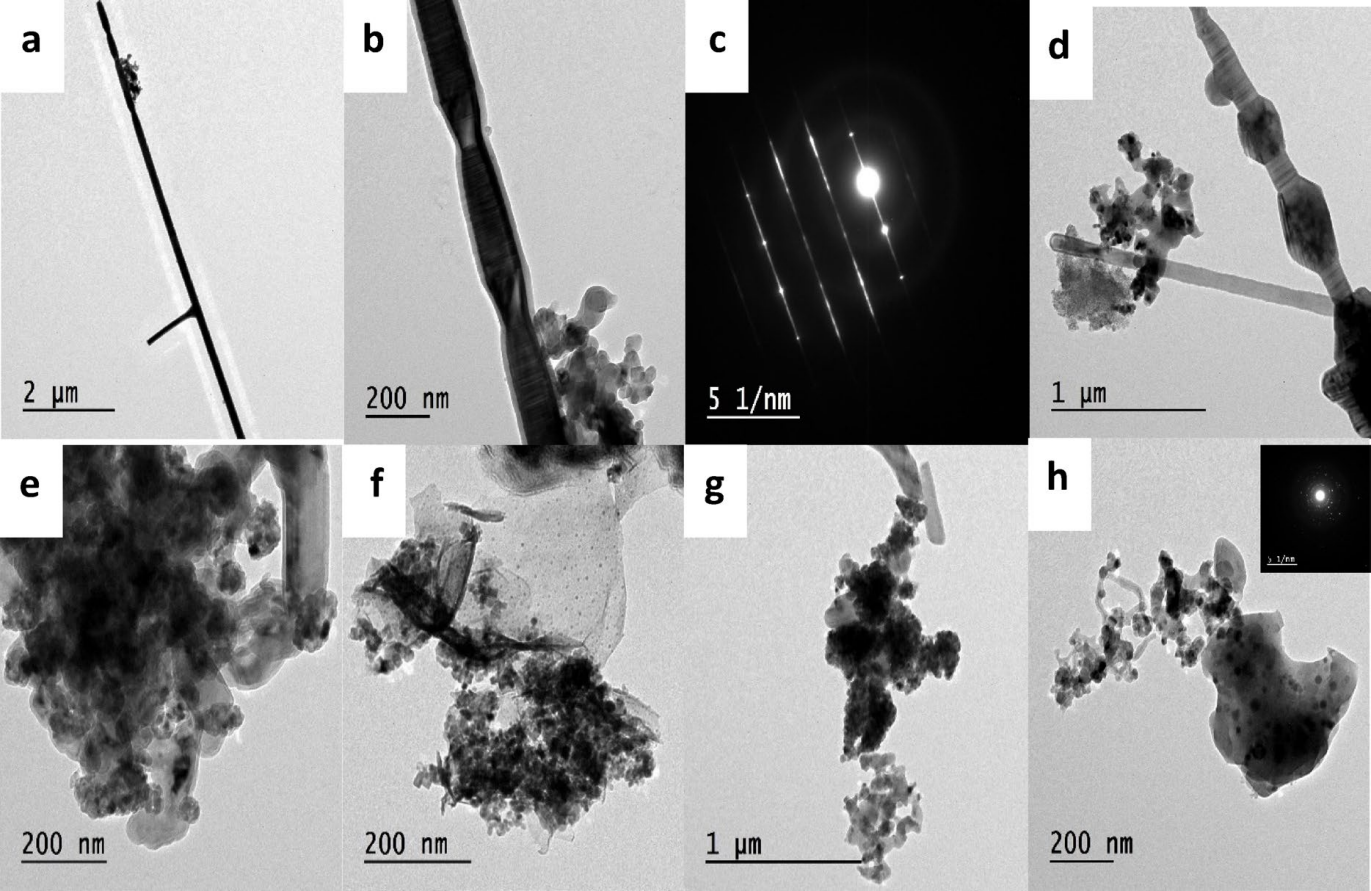
TEM images provide structural details where (**a**,**b**) reveal p-type SiC nanorods with uniform diameters and lengths, (**c**) crystalline SiC confirms with d-spacing values corresponding, (**d**) a bamboo-like morphology with hexagonal prism-shaped indicative of one-dimensional hierarchical structures, (**e**–**h**) display other nano-entities including dark spherical particles.

TEM images (Fig. 4a–h) provide further structural details. Figure 4a and b reveal p-type SiC nanorods with uniform diameters (~ 200 nm) and lengths up to tens of microns. The selected area electron diffraction (SAED) pattern (Fig. 4c) confirms crystalline SiC, with d-spacing values corresponding to the (111), (200), (220), and (311) planes. Figure 4d shows a bamboo-like morphology with hexagonal prism-shaped segments along the nanorods, indicative of one-dimensional hierarchical structures. These stacking faults, also observed in XRD patterns, may result from thermodynamically favorable structural rearrangements under internal stress^34^. The formation of SiC over Si₃N₄ is attributed to the higher nucleation rate of SiC under the given synthesis conditions. Figure 4e–h displays other nano-entities, including dark spherical particles (possibly β-Si₃N₄), brick-like TiC particles, carbon nitride sheets, and aggregated TiO₂ and SiO₂ nanoparticles. The SAED patterns (Fig. 4h inset) confirm their polycrystalline nature^35^.

####  Optical properties of the SiTiCNO nanocomposite

The optical properties of the synthesized SiTiCNO nanocomposite were investigated using UV–Vis diffuse reflectance spectroscopy (DRS), optical band gap analysis, and photoluminescence (PL) spectroscopy.

##### UV–Vis analysis

**Fig. 5:**
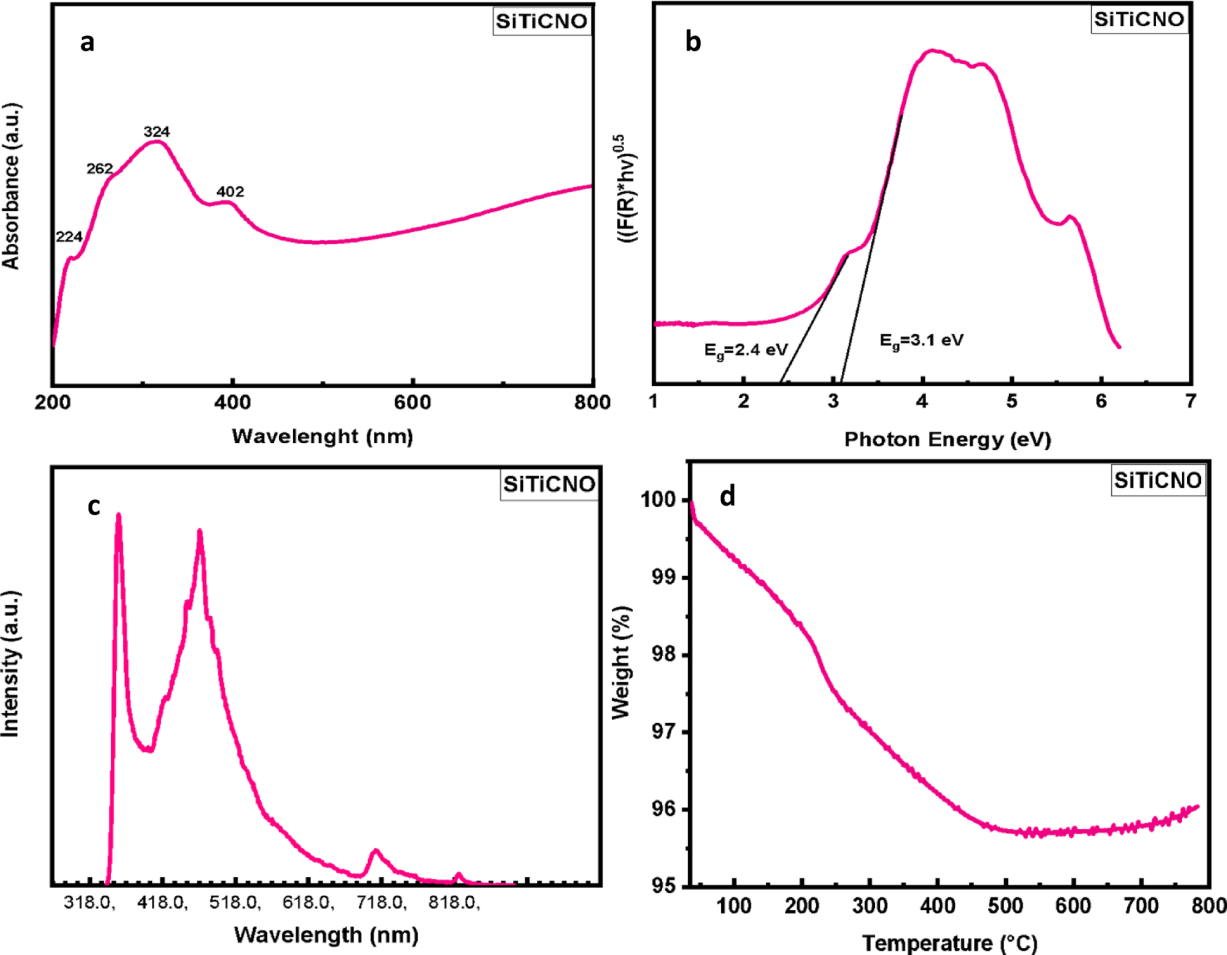
(**a**) The UV–Vis DRS spectrum of the sample, (**b**) The derived band gap energy for SiC, (**c**) The PL spectrum of the sample recorded at room temperature, (**d**) the thermogravimetric analysis (TGA) and derivative thermogravimetry (DTG) curves of the SiTiCNO nanocomposite.

Figure 5a displays the UV–Vis DRS spectrum of the sample. Four prominent absorption bands were observed at wave lengths approximately 224, 262, 324, and 402 nm. These bands are attributed to the individual components present in the nanocomposite. Specifically, the absorption band around 310 nm is ascribed to SiC, with a slightly lower wavelength compared to pure SiC^36^, indicating a minor quantum size effect or structural alteration. The absorption band near 324 nm corresponds to Si₃N₄ and SiO₂ phases, while those at 263 nm and 224 nm are attributed to TiC and TiO₂, respectively. The optical band gap (E_g_) was estimated using the Kubelka–Munk function and the Tauc plot method, as described by the equation^37^.

1 $${\left( {F\left( R \right)h\upsilon } \right)^{0.5}} = A\left( {h\upsilon - {E_g}} \right)$$

where F(R) is the Kubelka–Munk function, h is Planck’s constant, *υ* is the frequency of the incident photon, A is a constant, and E_g_​ is the optical band gap.

As shown in Fig. 5b, the derived band gap energy for SiC was approximately 2.4 eV, and for TiO₂ around 3.1 eV. These values are slightly higher than those reported for the corresponding bulk materials (2.39 eV for 3 C-SiC and 3.0 eV for TiO₂), suggesting quantum confinement effects and the presence of structural defects^38^.

##### Photoluminescence (PL) spectroscopy

Figure 5c shows the PL spectrum of the sample recorded at room temperature under excitation at 325 nm. Two distinct emission peaks were observed at 351 nm and 790 nm, exhibiting high-intensity emissions. These emissions are consistent with those previously reported for SiC nanowires and TiO₂ nanostructures, further indicating that the synthesized nanocomposite primarily consists of 1D SiC nanostructures (e.g., nanorods or nanowires).

The variation in PL emission positions compared to pure materials is attributed to differences in composition, structural defects, and nano-structuring. Defects such as stacking faults, twin boundaries, and polytypic growth in 1D SiC nanostructures—as well as quantum confinement contribute to the observed shifts in emission. These defects influence the density of states and introduce discrete energy levels within the band gap, which act as recombination centers for electron-hole pairs^39^. The PL emission energy (e.g., 2.64 eV corresponding to 470 nm) was slightly lower than the absorption edge, a phenomenon typically attributed to Stokes shift. The energy difference between the bulk 3 C-SiC and the nanowire form likely results from stacking faults and twin defects. These results align well with previous studies^40^, suggesting that visible emissions arise from electron-hole recombination between discrete energy levels within quantum wells formed by nanocrystalline domains embedded in amorphous or bulk-like regions^41^.

##### Thermogravimetric analysis (TGA)

Figure 5d shows the thermogravimetric analysis (TGA) and derivative thermogravimetry (DTG) curves of the SiTiCNO nanocomposite. The measurement was conducted up to 800 °C at a heating rate of 10 °C/min under a nitrogen atmosphere. A total weight loss of approximately 4% was observed up to 500 °C, with no significant change beyond this temperature, indicating excellent thermal stability of the sample. The observed weight loss is primarily due to the evaporation of surface-bound moisture and the slight decomposition of residual free carbon and minor components such as Si₃N₄^42^.

##### Surface area and porosity

**Fig. 6:**
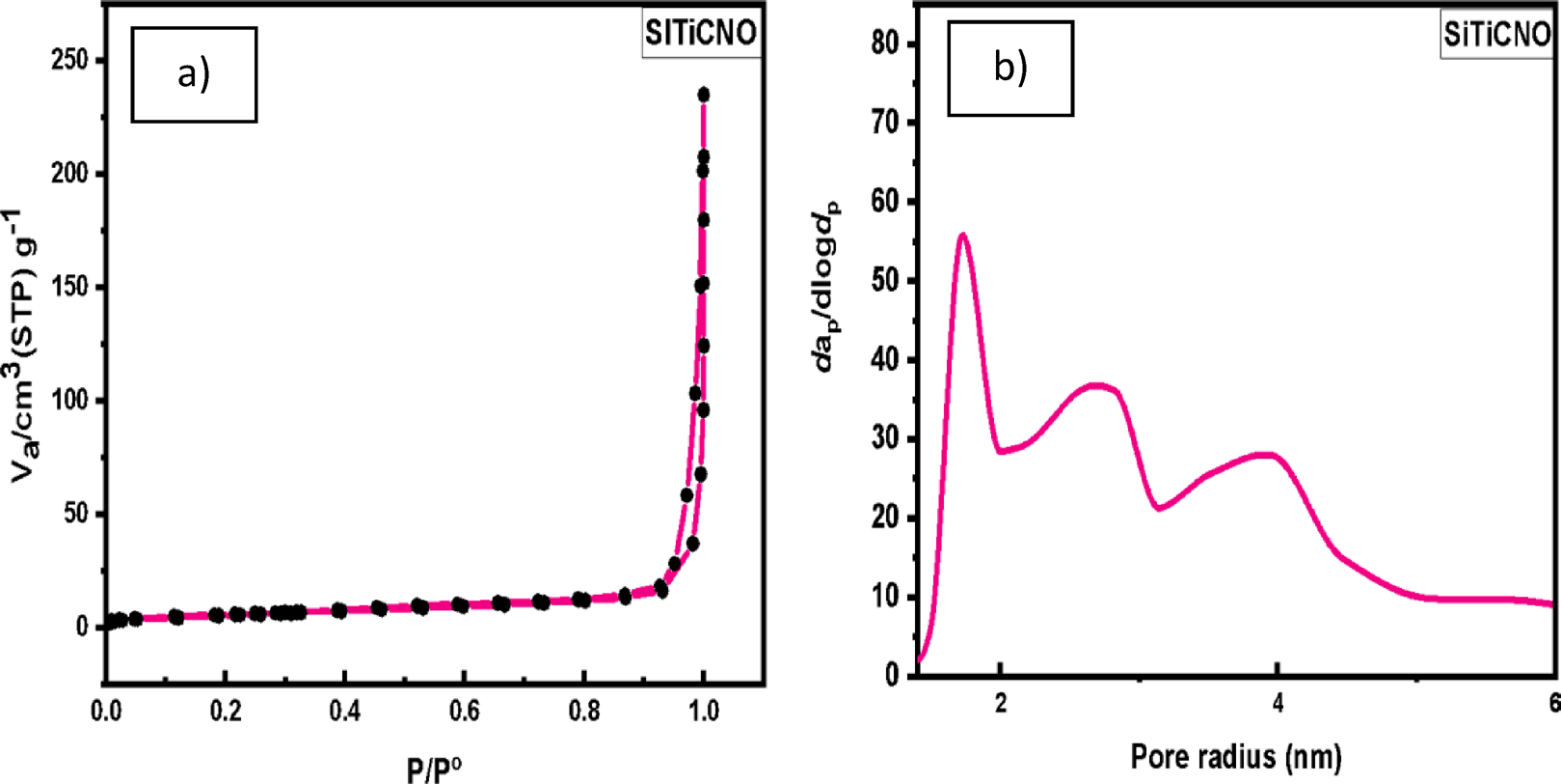
(**a**) The nitrogen display adsorption–desorption isotherm and (**b**) corresponding pore size distribution.

Figure 6a and b display the nitrogen adsorption–desorption isotherm and corresponding pore size distribution, respectively. The isotherm is classified as type IV according to IUPAC, with a clear hysteresis loop indicating the presence of mesoporous structures. The measured Brunauer–Emmett–Teller (BET) surface area of the sample was approximately 20.4 m^2^/g. The adsorption curve at low relative pressure suggests the presence of micropores, while the pore size distribution (Fig. 6b) reveals the existence of both micropores and mesopores in the range of 1.0–4.0 nm^35^.

### Electrodeposition of Ni@SiTiCNO

#### The coating thickness and surface morphology

**Fig. 7:**
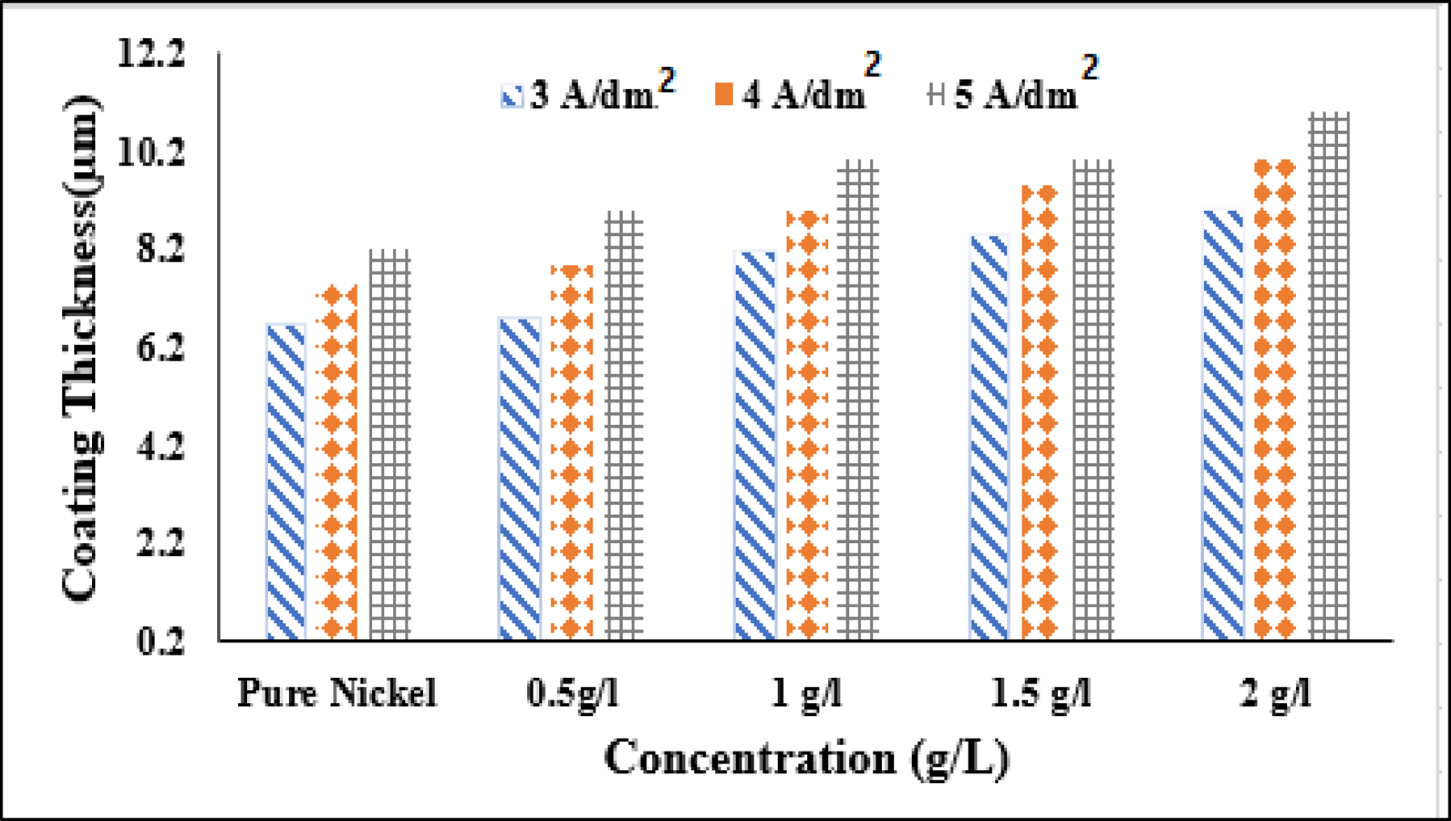
The influence of SiTiCNO nanoparticle concentrations on the thickness of Ni layer deposited at different current density for 30 min.

The layer thickness of the nanocomposite Ni@SiTiCNO and traditional Ni (pure Ni) electrochemically formed at various current densities (C.D.) and SiTiCNO nano-particle contents is displayed in Fig. 7. According to Faraday’s rule of electrolysis, which states that higher current densities result in higher deposition rates, layer thickness rises as C.D. increases across all contents. For the pure nickel coating, a steady rise in thickness is observed from 3 A/dm^2^ to 5 A/dm^2^. Interestingly, at a low particle concentration (0.5 g/L), a reduction in coating thickness is observed at 3 A/dm^2^ and 4 A/dm^2^, likely due to inadequate particle incorporation or disturbance of the electro crystallization process. However, at higher concentrations (≥ 1 g/L), a progressive increase in thickness is noted, especially at 5 A/dm^2^, indicating improved particle co-deposition and better matrix formation. The maximum thickness (~ 13 μm) was achieved at 2 g/L and 5 A/dm^2^, highlighting the synergistic effect of sufficient particle availability and enhanced electrochemical driving force^43^. This enhances performance by improving the uniformity, morphology, and interfacial properties of the deposited nanocomposite, leading to better structural integrity, mechanical strength, and electrochemical activity for various device applications.

**Fig. 8:**
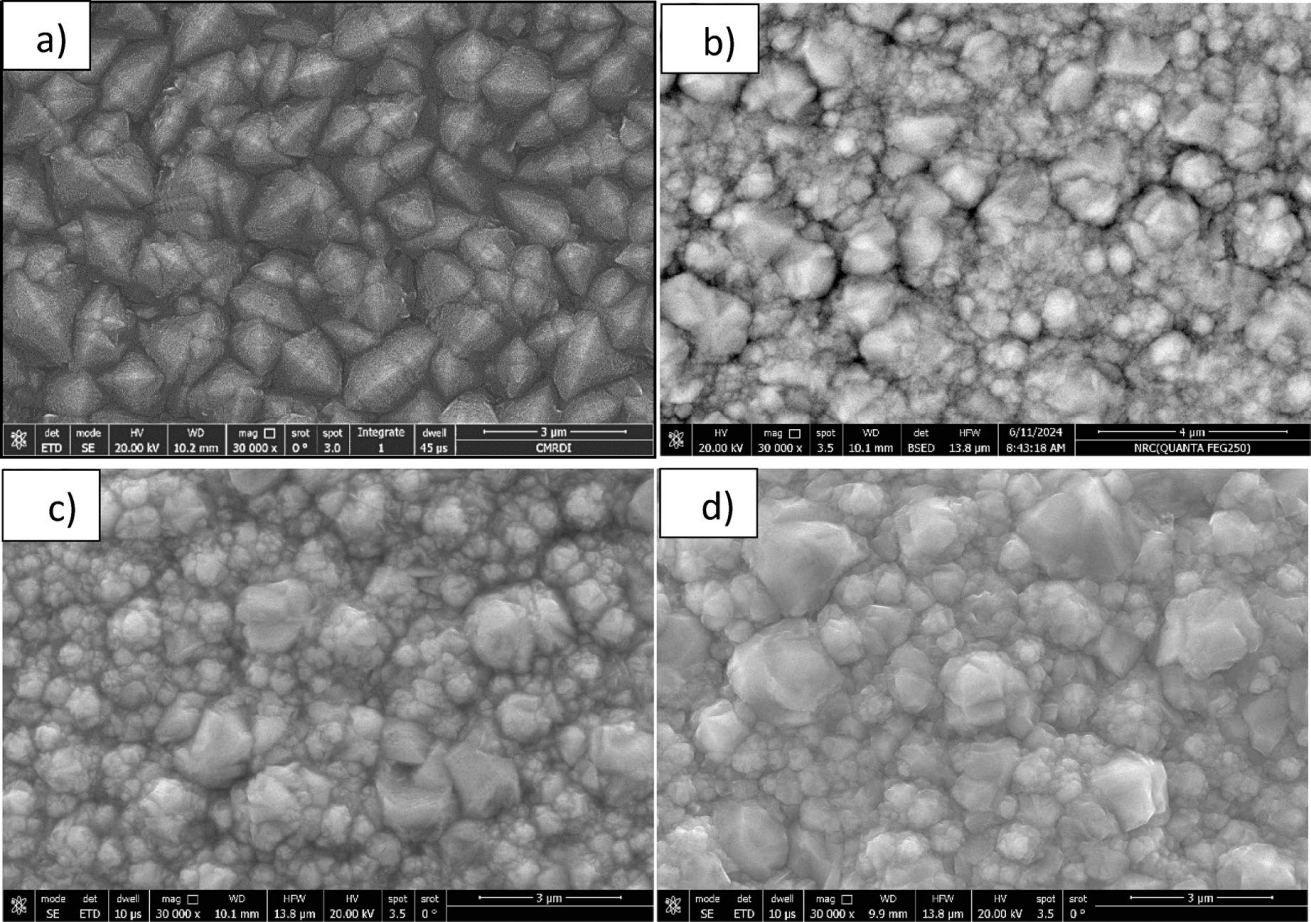
SEM images of Ni and Ni@SiTiCNO nanocomposites deposited at 5 A/dm^2^ at 30 min and different SiTiCNO nanoparticles where, (**a**) Pure Ni layer, and at adding, (**b**) 1 g/L, (**c**) 1.5 g/L, and (**d**) 2 g/L SiTiCNO nanoparticles.

The surface topography of Ni@SiTiCNO nanocomposite coatings and pure Ni coatings deposited with different C.D. and nano SiTiCNO contents can be seen in Fig. 8. The SEM image (Fig. 8a) demonstrated that the perfect Ni crystal shape was achieved, and after adding SiTiCNO, the morphology was changed. The morphology of the Ni@SiTiCNO nanocomposite changed and formed a crystalline-like structure, clearly indicating that the SiTiCNO nanoparticles have been successfully embedded in the Ni matrix. Pure Ni clearly showed the large size of nickel grains and their extent of bonding, and it was evident that with the increase in the concentration of the reinforcement, the branching and cohesion of the grains increased.

**Fig. 9:**
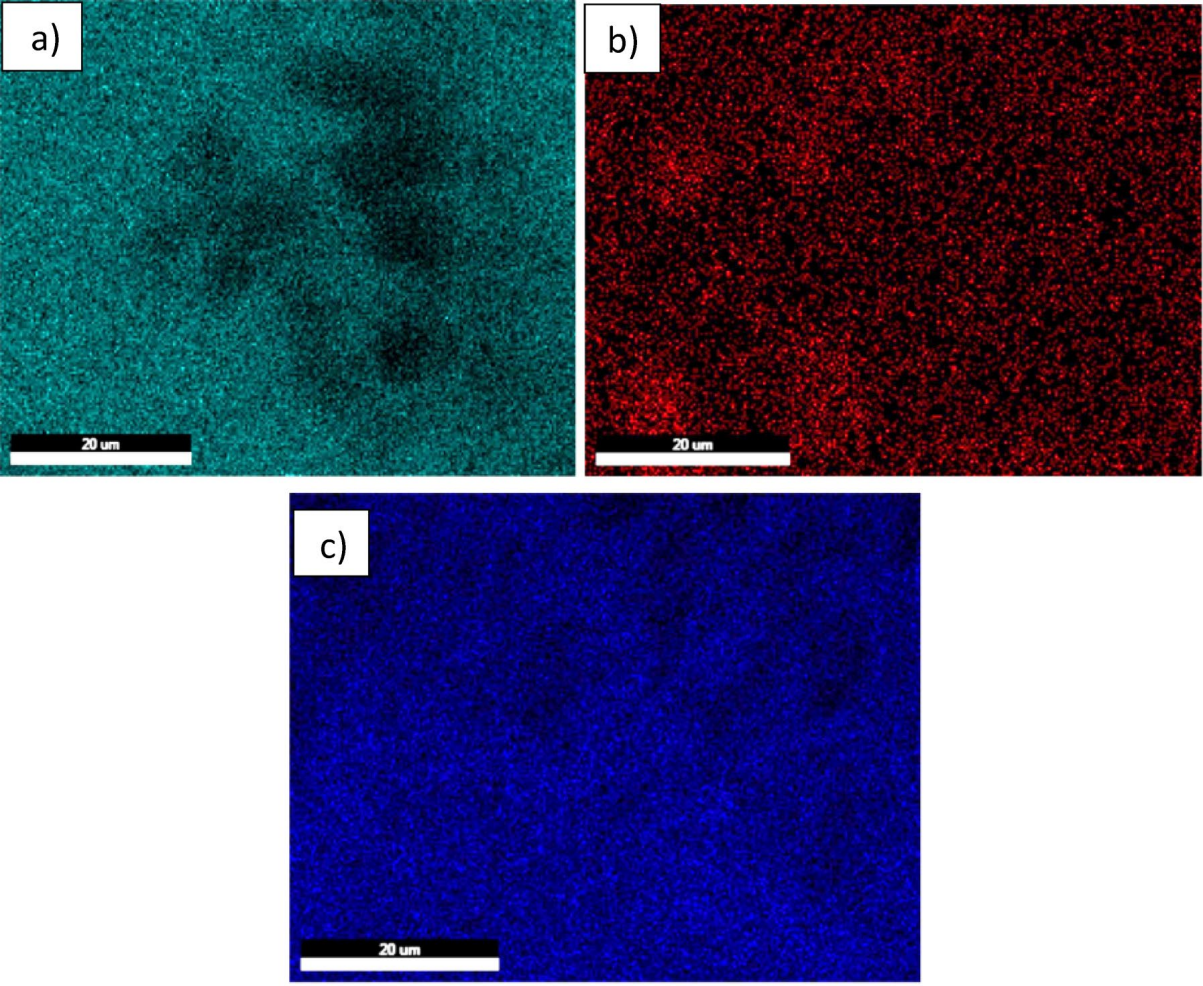
Map images of Ni@SiTiCNO nanocomposite deposited at 5 A/dm^2^ at 30 min and 2 g/L SiTiCNO, where, (**a**) Ni, (**b**) Si, and (**c**) Ti.

Mapping analysis technique was used to illustrate the incorporation of SiTiCNO nanoparticles with the nickel layer, as is shown in Fig. 9. The term “mapping analysis” describes the spatial characterization of the chemical constituents and/or phases found in nickel-based composite coatings, which are typically made from SiTiCNO ceramic precursors and comprise silicon (Si), titanium (Ti), carbon (C), nitrogen (N), and oxygen (O). SiTiCNO nanoparticles were effectively inserted into the Ni matrix, as demonstrated by the Si and Ti images in Fig. 9b and c.

**Fig. 10:**
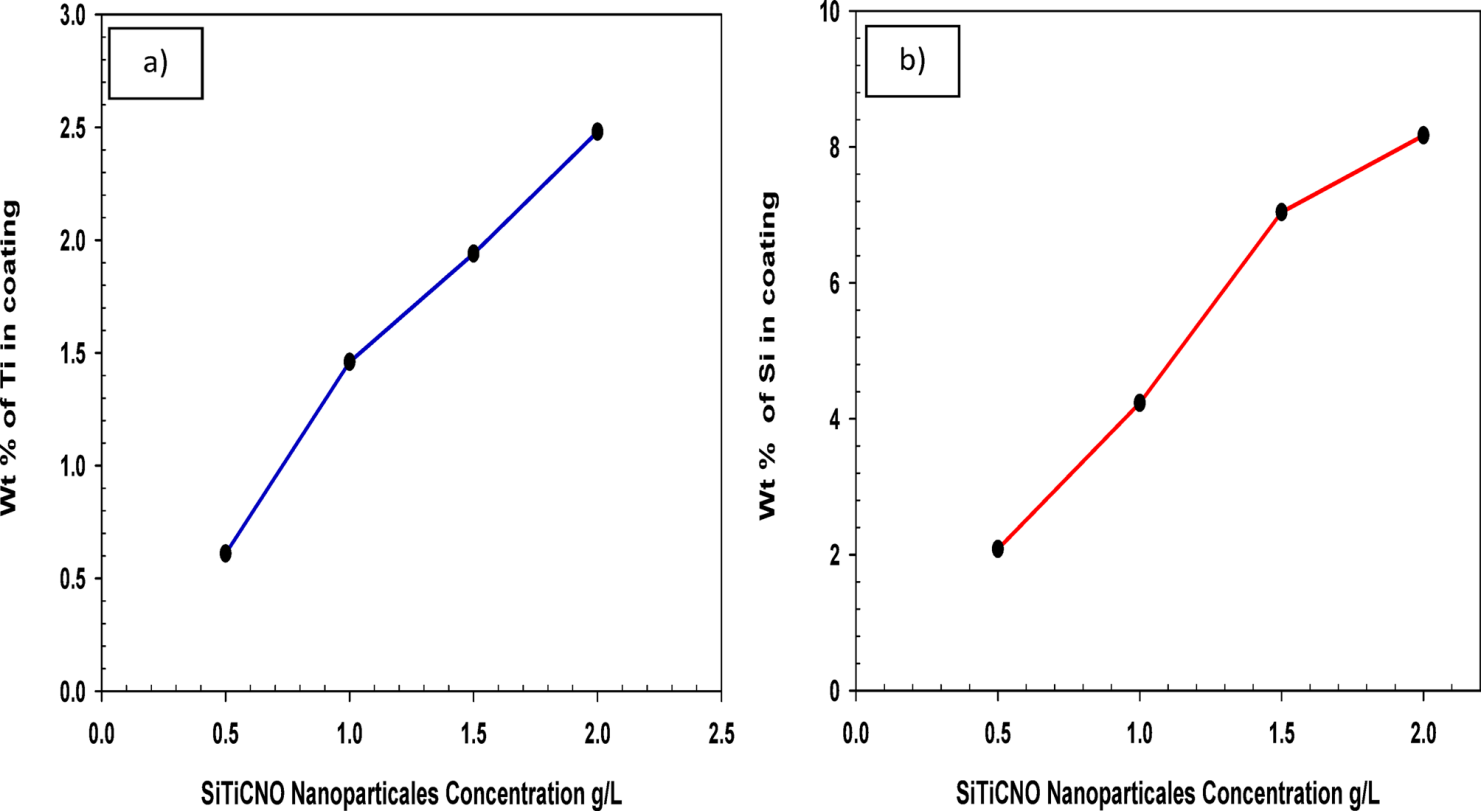
The influence of SiTiCNO nano powder concentrations in the plating electrolyte on (**a**) Ti, (**b**) Si wt% in the nickel matrix.

For examining elemental makeup and distribution of multi-phase coatings, Energy Dispersive X-ray Spectroscopy (EDX) is an effective method. Both qualitative and semi-quantitative characterization of the coating’s structure are made possible by EDX when combined with SEM (Scanning Electron Microscopy). The impact of SiTiCNO contents in the depositing electrolyte upon the resulting weight% of SiTiCNO nanoparticles incorporated into the nickel matrix was systematically investigated using EDX. EDX analysis confirmed the presence of Ni as the primary matrix element, with Si, Ti, while C, N, and O cannot be confirmed, may be due to their low percentage. Elemental mapping showed a uniform distribution of the ceramic constituents throughout the nickel matrix (Si and Ti), suggesting effective particle dispersion and incorporation during electrodeposition. As illustrated in Fig. 10a and b, the experimental results reveal a direct correlation between the concentration of suspended particles in the electrolyte and their subsequent co-deposition within the Ni matrix (Si and Ti). The data demonstrate that the weight% of Si and Ti through the composite coating rises markedly with increasing SiTiCNO contents in the plating solution, attaining an optimal value at 2 g/L SiTiCNO. According to the findings, the weight% of Si and Ti via the composite coating rises noticeably as the electrolyte concentrations rise, reaching its ideal value at 2 SiTiCNO.

Several theoretical frameworks have been presented to clarify the basic mechanisms driving the integration of nanoparticles into the Ni matrix during nanocomposite electrodeposition. To demonstrate the impact of particle surface charge on the deposition process, hypothesized that the inclusion of fine particles is primarily controlled by their transport behavior and electrokinetic properties within the plating electrolyte^1^. Adsorbed ionic species provide solid particles in the electrolyte with an electrolytic charge, the sign and magnitude of which are measured by the zeta potential (ζ).

**Fig. 11:**
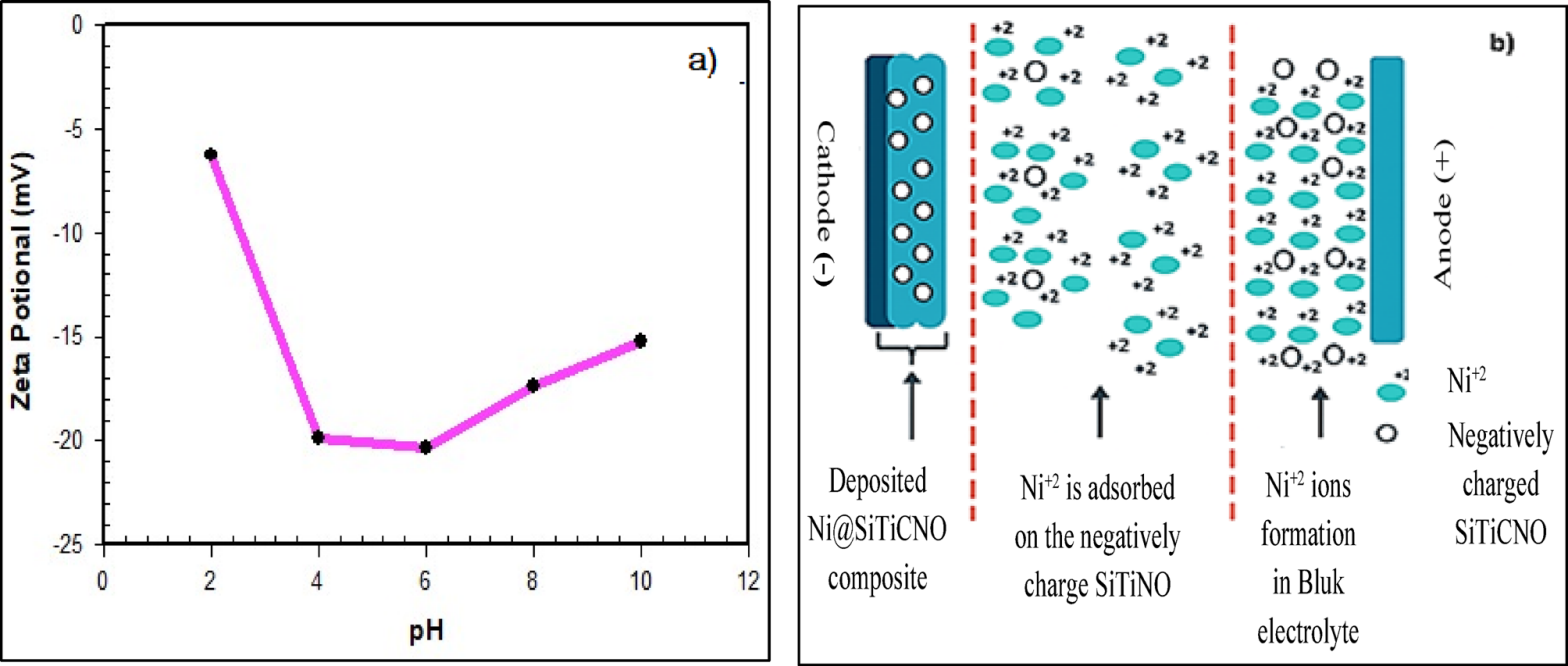
(**a**) Zeta potential of SiTiCNO nanoparticles at different pH values and (**b**) The proposed mechanism electrodeposition production of Ni@SiTiCNO nano composite coatings.

A key element in the successful electrodeposition creation of Ni@SiTiCNO composite coatings is the surface charge behavior (zeta potential) of the dispersed SiTiCNO nano-particles in the electrolyte solution. During the electrochemical process, the zeta potential directly controls the ceramic phase’s colloidal stability, mobility, and codeposition efficiency. Characterization of SiTiCNO particles revealed a consistently negative ζ value across the pH range 2 to 11 in dilute plating electrolyte (Fig. 11a). Consequently, the proposed mechanism involves negatively charged SiTiCNO particles adsorbing positively charged Ni^2+^ ions. These ion-adsorbed particles are then transported cathodically, where reduction of the adsorbed metal ions to Ni atoms integrates them into the growing deposit, resulting in the entrapment of SiTiCNO particles within the Ni matrix, as schematically depicted in Fig. 11b.

### The corrosion test

**Fig. 12:**
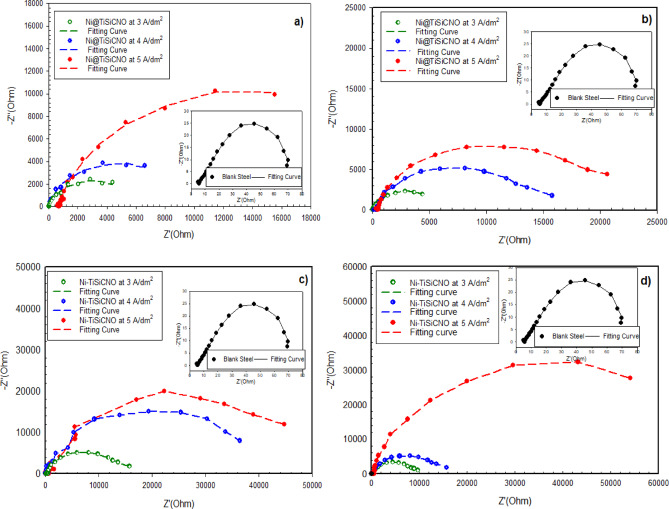
Nyquist (NQ diagrams) of impedance spectra for uncoated steel, Ni-coated steel and Ni@SiTiCNO nanocomposite-coated steel at different current density (3–5 A/dm^2^) and using different concentrations of Ni@SiTiCNO where, (**a**) 0.5 g/L, (**b**) 1 g/L, (**c**) 1.5 g/L, (**d**) 2 g/L deposited in 3.5% urea solution.

**Fig. 13:**
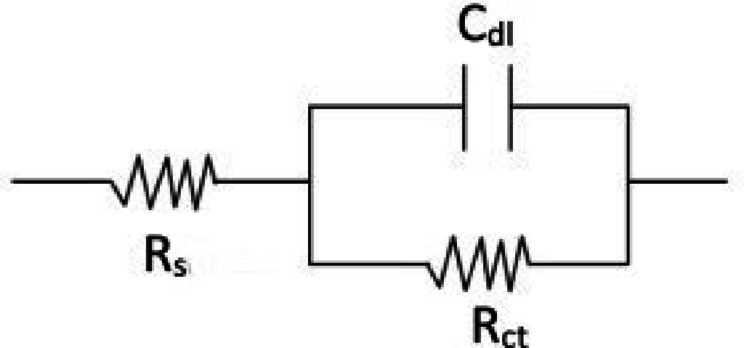
The equivalent electrical circuit (EEC) model.

 The Nyquist (NQ) diagrams of unprotected steel, steel protected by Ni, and steel protected by Ni@SiTiCNO nanocomposite at varied SiTiCNO concentrations (0.5–2 g/L) and operated C. D. (3–5 A/dm^2^) in 3.5% urea solution are shown in Fig. 12a–d. Several kinetic parameters, including R_s_ (solution resistance), R_ct_ (charge transfer resistance), and C_dl_ (double-layer capacitance), were obtained by fitting the best equivalent circuit model to the data to resolve the NQ diagram using the electrical equivalent circuit (EEC), as seen in Fig. 13. The comparable circuit model that best matched the obtained impedance data was the R_s_(R_ct_C_dl_) circuit, which is a one-time constant (one capacitive loop) (Fig. 13). The semicircular diameter in the NQ diagrams has been used to clarify the surface’s resistance against the transfer of charge (R_ct_). Figure 12 illustrates how the addition of SiTiCNO at varying concentrations affects the transfer of charge (R_ct_) and how the coating layer’s resistance improves at all SiTiCNO concentrations compared to Ni-coated and uncoated steel. Additionally, Fig. 12a–d show a similar effect at present density values. With a concentration of 2 g/L SiTiCNO and operating at different currents than the other sample, the Ni@SiTiCNO nanocomposite coating exhibits the largest semicircular diameter.

As shown in Fig. 12a, as the current density increased, the charge transfer resistance (R_ct_) at a concentration of 0.5 g/L increased until it reached 13,510 Ω, and in Fig. 12b, as the nanocomposite content rose to 1 g/L and current density rose, the (R_p_) increased to 20,620 Ω. In Fig. 12c, as the content rose to 1.5 g/L and the current density was 5 A/dm^2^, the R_ct_ reached 44,660 Ω. Meanwhile, in Fig. 12d, when the nanocomposite concentration reached 2 g/L with the use of different current densities (3–5 A/dm^2^), the charge transfer resistance (R_ct_) increased to 54,150 Ω at 5 A/dm^2^. The diameter grows as the % of SiTiCNO nanoparticles increases until 2 g/L, according to the NQ diagrams. The authors may infer from the impedance data that, in comparison to steel coated with Ni film, the corrosion resistance rises as the % of SiTiCNO nanoparticles increases and the value of C_dl_ drops. This was explained by an electrical double layer becoming thicker. Therefore, low-capacity layers provide the best corrosion protection since they are less active and hence more corrosion-resistant.

Data extracted from the generated heatmap (Fig. S2) demonstrated a significant enhancement in corrosion resistance with increasing both current density and SiTiCNO nanoparticles concentration. The charge transfer resistance (R_ct_) increased dramatically from 550 Ω·cm^2^ for the uncoated steel sample to 54,150 Ω·cm^2^ for the sample coated with Ni and 2 g/L of SiTiCNO at 5 A/dm^2^. Simultaneously, the double-layer capacitance (C_dl_) was notably reduced to 9.98 × 10⁻⁵ F, indicating a surface with fewer defects and a more compact structure. These improvements suggest that the incorporation of SiTiCNO nanoparticles effectively fills micro-voids in the Ni matrix, reduces porosity, and enhances electrochemical stability. Therefore, employing higher concentrations of SiTiCNO under elevated current densities appears to be a promising strategy for developing high-performance anti-corrosive coatings for industrial applications exposed to aggressive environments. Moreover, the R_ct_ greatest value, indicating the highest corrosion resistance, according to the impedance data displayed in Table 3. This indicates that the coating’s resistance to corrosion is increased when SiTiCNO nanoparticles are added to a Ni matrix. Additionally, the addition of SiTiCNO nanoparticles to the Ni matrix on the steel surface reduced the penetration of oxygen to the steel base, which in turn reduced the movement of ions and electrons among the metallic surface and the corrosive fluid. Furthermore, by filling the voids and fissures on the surface of Ni coatings, the nanoparticles in the Ni matrix act as physical obstacles to the corrosion procedure^44^.

**Fig. 14:**
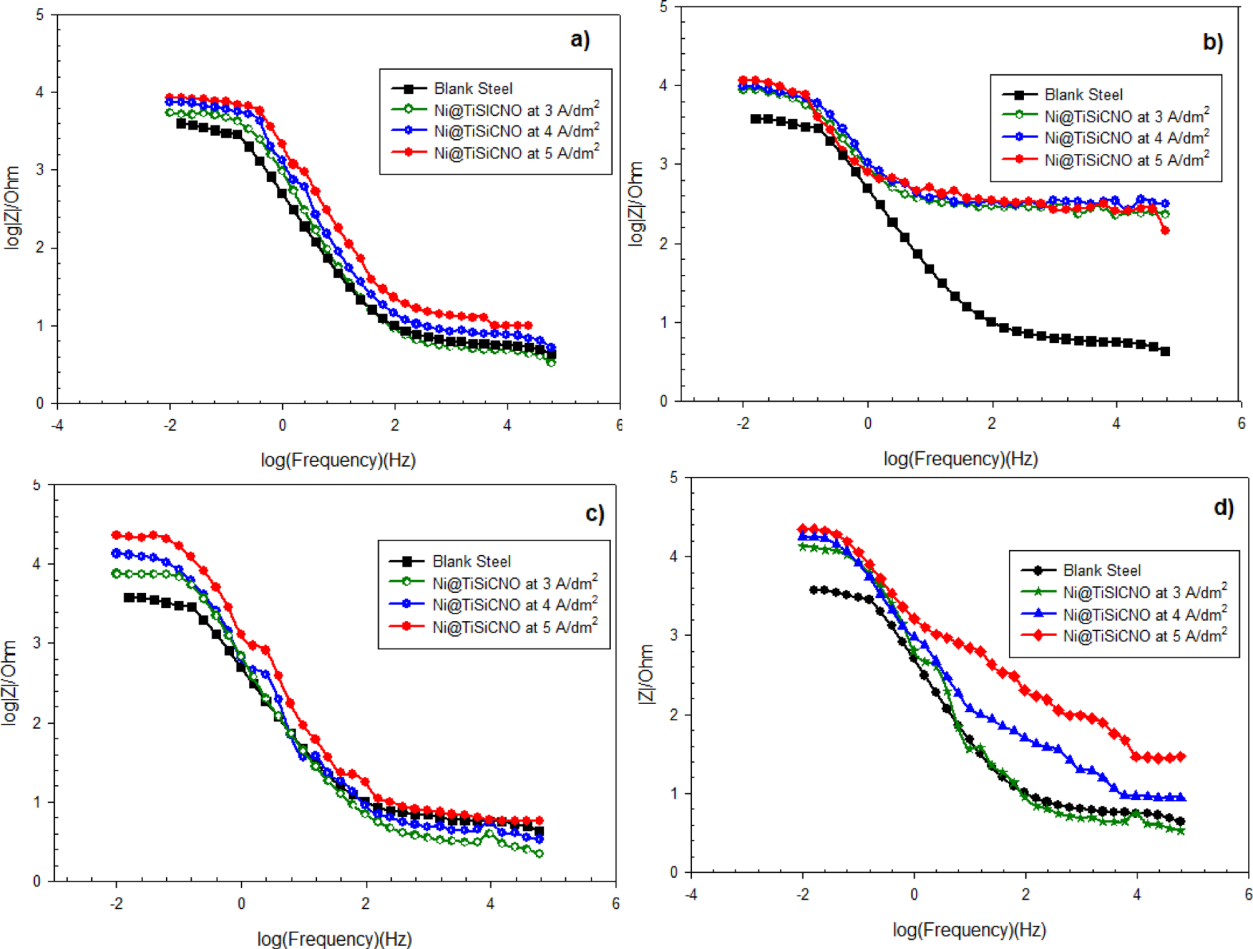
Bode plots of impedance spectra for uncoated steel, Ni-coated steel and Ni@SiTiCNO nanocomposite-coated steel at different current density (3–5 A/dm^2^) and using different concentrations of Ni@SiTiCNO where, (**a**) 0.5 g/L, (**b**) 1 g/L, (**c**) 1.5 g/L, (**d**) 2 g/L deposited at different current density.

The Bode plot and the phase angle are two crucial factors that are frequently utilized to understand findings in the impedance data. Bode plot shows how the total impedance of a system varies with frequency. This plot illustrates the relationship between frequency (on a logarithmic scale, usually in Hz) and the impedance magnitude, typically measured in ohms or log (Ohm). Figure 14a–d displays the Bode diagrams of the unprotected steel, traditional Ni-coated stee, and Ni@SiTiCNO nanocomposite-coated steel using various C. D. (3–5 A/dm^2^) and different SiTiCNO concentrations (0.5–2 g/L). The various graphs show that the impedance magnitude clearly drops at lower frequencies with blank steel, indicating a low resistance to corrosion. While, the impedance magnitude increases as the current density and the concentration of SiTiCNO nano powders increase, indicating an improvement in corrosion resistance. The coating exhibits the maximum impedance magnitude at a high concentration of nanopowder (2 g/L) and a high current density of 5 A/dm^2^, which may provide the best corrosion protection and the best resistance to electrochemical processes.

**Table 3:** The EIS parameter of steel uncoated, steel coated with pure Ni and Ni-SiTiCNO nanocomposite coatings from watt’s bath at various current densities and Ni@SiTiCNO nanoparticles contents in 3.5% urea solution.

Sample name	$\:{R}_{s,}$	$\:{R}_{ct,}\left(\varOmega\:c{m}^{2}\right)$	$\:C,F$
Blank Steel	23.80 ± 0.48	550 ± 28	1.483 ± 0.104
$\:Ni\:coated\:steel\:at\:3\:A/d{m}^{2}$	78.76 ± 1.58	2710 ± 136	2.66^− 4^ ± 1.86^− 5^
Ni-0.5 g/L Ni@SiTiCNO	86.86 ± 1.74	3119 ± 156	2.84^− 4^ ± 1.99^− 5^
Ni-1 g/L Ni@SiTiCNO	115.0 ± 2.30	4379 ± 219	1.07^− 4^ ± 7.49^− 6^
Ni-1.5 g/L Ni@SiTiCNO	91.53 ± 1.83	9710 ± 486	1.10^− 4^ ± 7.70^− 6^
Ni-2 g/L Ni@SiTiCNO	97.96 ± 1.96	13,760 ± 688	2.84^− 4^ ± 1.99^− 5^
$\:Ni\:coated\:steel\:at\:4\:A/d{m}^{2}$	97.34 ± 1.95	5701 ± 285	1.23^− 5^ ± 8.61^− 7^
Ni-0.5 g/L Ni@SiTiCNO	196.6 ± 3.93	6605 ± 330	2.10^− 4^ ± 1.47^− 5^
Ni-1 g/L Ni@SiTiCNO	169.7 ± 3.39	13,000 ± 650	1.17^− 4^ ± 8.19^− 6^
Ni-1.5 g/L Ni@SiTiCNO	222.0 ± 4.44	15,760 ± 788	1.05^− 4^ ± 7.35^− 6^
Ni-2 g/L Ni@SiTiCNO	221.0 ± 4.42	20,460 ± 1023	2.04^− 4^ ± 1.43^− 5^
$\:Ni\:coated\:steel\:at\:5\:A/d{m}^{2}$	380.2 ± 7.60	13,460 ± 673	1.10^− 4^ ± 7.70^− 6^
Ni-0.5 g/L Ni@SiTiCNO	420.0 ± 8.40	13,510 ± 676	1.24^− 5^ ± 8.68^− 7^
Ni-1 g/L Ni@SiTiCNO	510.8 ± 10.22	20,620 ± 1031	1.16^− 4^ ± 8.12^− 6^
Ni-1.5 g/L Ni@SiTiCNO	678.7 ± 13.57	44,760 ± 2238	8.21^− 5^ ± 5.75^− 6^
Ni-2 g/L Ni@SiTiCNO	798.4 ± 15.97	54,150 ± 2708	9.98^− 5^ ± 6.99^− 6^

**Fig. 15:**
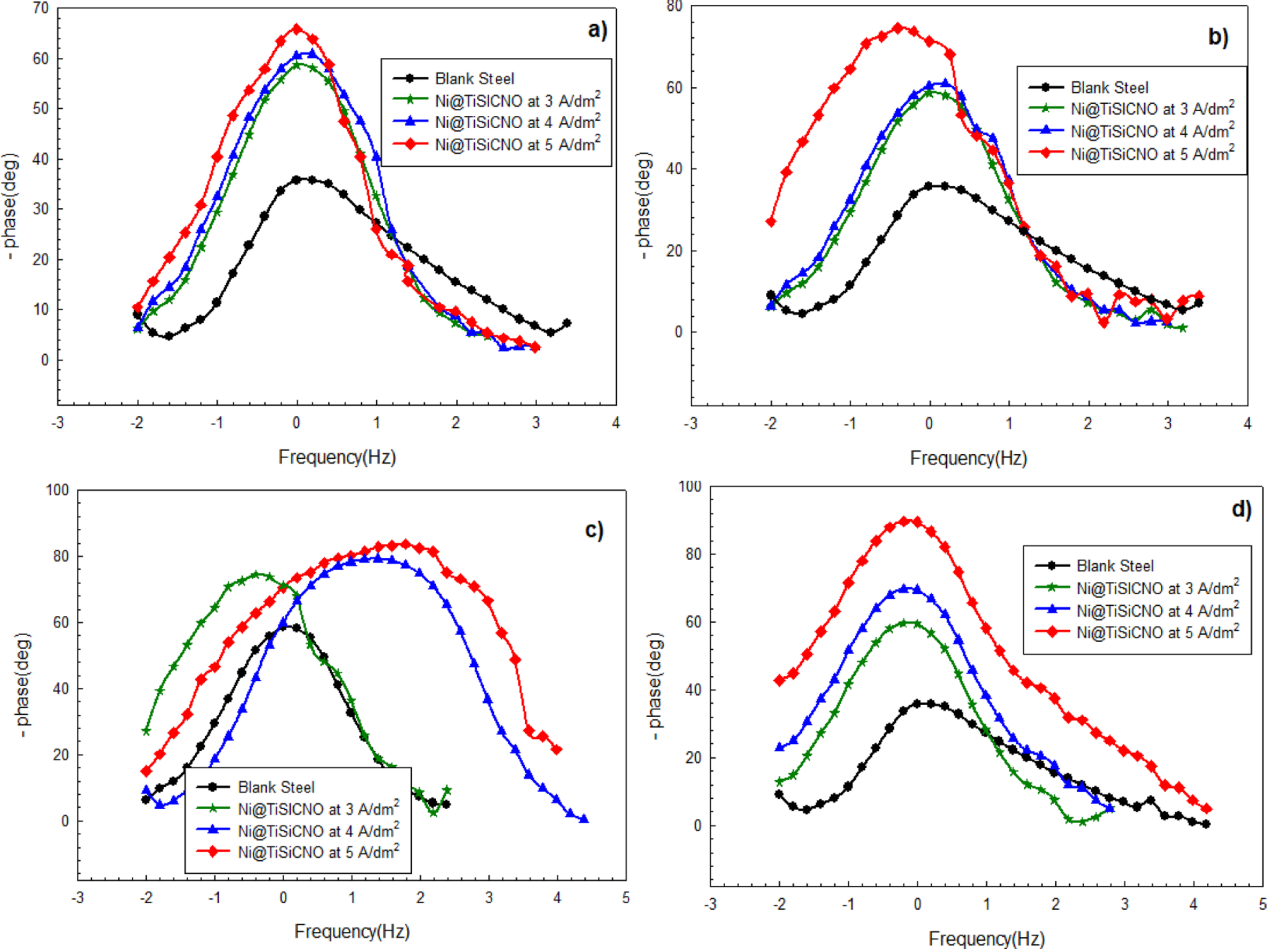
Phase angle plots of impedance spectra for uncoated steel, Ni-coated steel and Ni@SiTiCNO nanocomposite-coated steel at different current density (3–5 A/dm^2^) and using different concentrations of Ni@SiTiCNO where, (**a**) 0.5 g/L, (**b**) 1 g/L, (**c**) 1.5 g/L, (**d**) 2 g/L deposited at different current density.

Generally, the phase angle reveals the capacitive behavior of a system. The higher the phase angle, the more capacitive, which means better barrier properties and improved corrosion resistance. Moreover, a broader phase angle peak means a more stable passive layer/coating. At the same time, multiple peaks mean more than one time constant (coating layer and double layer^45^. Figure 15a–d show phase angle vs. frequency curves from EIS measurements. These curves help evaluate the corrosion protection performance of Ni@SiTiCNO coatings on steel substrate. Furthermore, all coated samples at various current densities and SiTiCNO nanoparticle concentrations show higher phase angles compared to uncoated steel, especially at mid frequencies, indicating capacitive behavior and suggesting effective corrosion protection. The peak position shifts slightly to the right as the current density increases, and the magnitude also rises. The best performance is seen at 5 A/dm^2^ and 2 g/L nanoparticles; the highest and sharpest peak indicates better coating uniformity or a stronger barrier effect. This indicates that the coating’s resistance to corrosion is increased when SiTiCNO nanoparticles are added to a Ni matrix. Additionally, the addition of SiTiCNO nanoparticles to the Ni matrix on the steel surface reduced the permeability of oxygen to the steel surface, which in turn reduced the movement of ions and electrons among the metallic surface and the corrosive fluid. Moreover, the SiTiCNO nanoparticles dispersion in the composite coatings promoted homogenous corrosion by reducing localized corrosion.

**Fig. 16:**
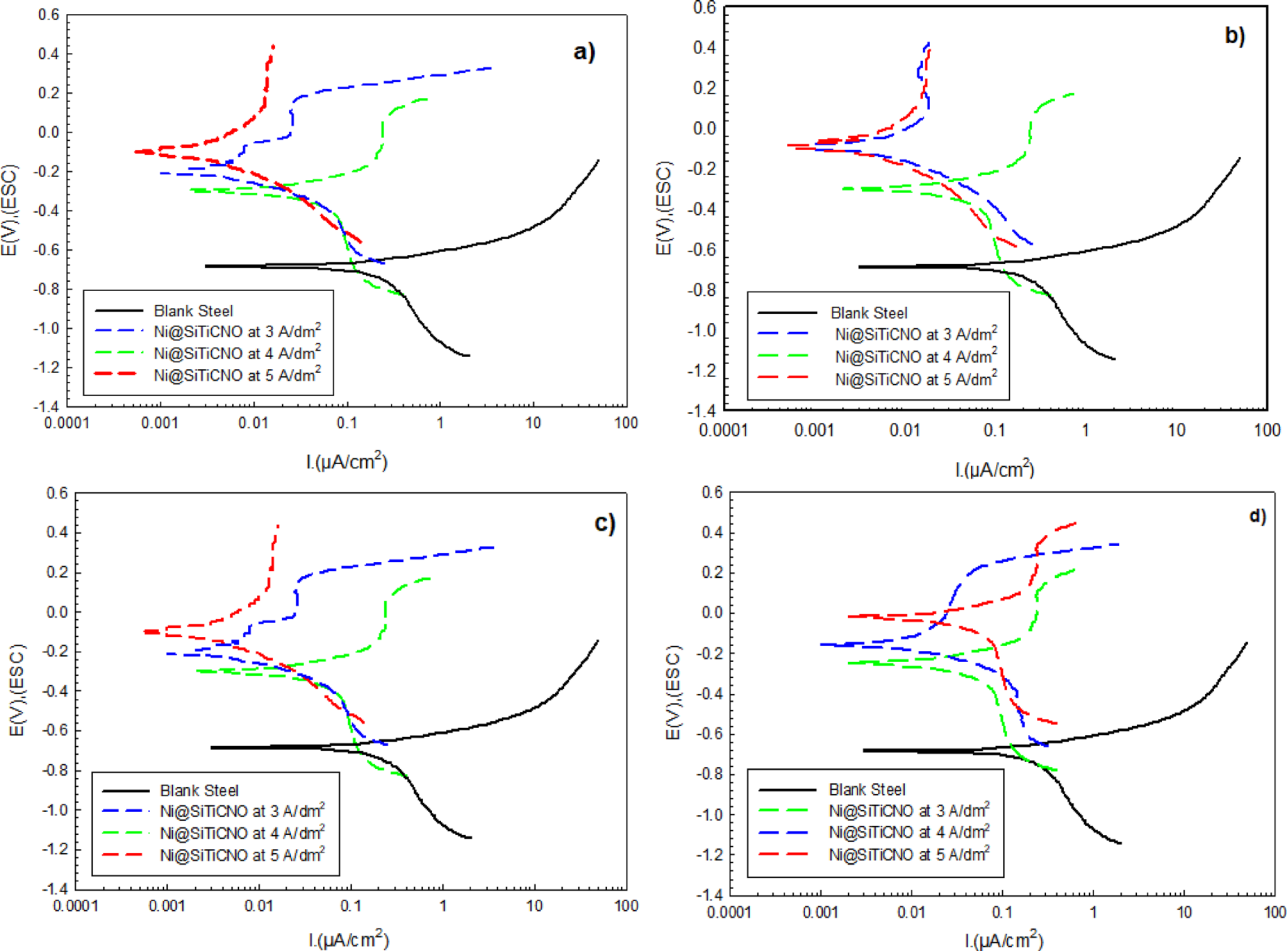
The potentiodynamic polarization in 3.5% urea solution for uncoated steel, Ni-coated steel and Ni@SiTiCNO nanocomposite-coated steel at different current density (3–5 A/dm^2^) and using different concentrations of SiTiCNO where, (**a**) 0.5 g/L, (**b**) 1 g/L, (**c**) 1.5 g/L, (**d**) 2 g/L deposited at different current density.

For the same samples under investigation, Tafel polarization curves (Fig. 16) were analyzed to validate the EIS findings. Table 4 provides a summary of the associated electrochemical characteristics, such as corrosion potential (E_corr_), corrosion current density (I_corr_), polarization resistance (R_p_), and corrosion rate (mm/a). The polarization behavior was assessed for coatings deposited at various current densities (3–5 A/dm^2^) and concentrations of SiTiCNO nanoparticles (0.5–2 g/L), as shown in Fig. 16; Table 4. The impact of current densities and nanoparticle concentration on the electrochemical response was observed. The results showed that deposition at 4 and 5 A/dm^2^ produced more positive electrochemical performances compared to 3 A/dm^2^. This indicates that the electrochemical performance changes as the current increases.

The effect of nanoparticle concentrations on the electrochemical response was observed as the concentration varied. The results indicated that adding 2 g/L of nanoparticles produced better electrochemical performance compared to traditional nickel coating and other coating samples. This suggests that the electrochemical performance improves with an increase in nanoparticle concentration. Ni@SiTiCNO nanocomposite coating deposited at 5 A/dm^2^ with 2 g/L nanoparticle content exhibited the lowest corrosion rate of 0.0085 mm/a compared with the conventional Ni layer (0.022 mm/a), indicating superior corrosion resistance. This suggests that the anodic and cathodic reactions of the corrosion process were lessened by the addition of SiTiCNO. The corrosion potential also changed to less negative values. This might be a result of the SiTiCNO inhibiting hydrogen production. It is evident that the corrosion rate and corrosion current density significantly decreased as the concentration of SiTiCNO increased. Thus, it can be concluded that SiTiCNO can shield the coated steel’s surface from corrosive ion attacks in corrosive environments^46^.

Moreover, dispersed ceramic particles (SiTiCNO) in the Ni matrix produce a more compact, less porous, and tortuous microstructure, and the barrier effect is believed to be the demonstrated mechanism. The particles fill surface defects and micro-voids. They interrupt the direct pathways for electrolyte penetration. They reduce the real electrochemically active surface area. This mechanism reduces ionic diffusion towards the metal/electrolyte interface, which slows the metal’s dissolution and lowers i_corr_. makes the surface more stable, which causes E_corr_ to move in a more noble direction. These results confirm the synergistic role of nanoparticle content in optimizing the anticorrosive performance of Ni@SiTiCNO composite coatings with current density.

**Table 4:** A summary of the associated electrochemical characteristics for uncoated steel, Ni-coated steel, and Ni@SiTiCNO nanocomposite-coated steel at different current density and using different concentrations of SiTiCNO.

Sample name	$\:{E}_{corr}\left(\mathrm{V}\right)$	$\:{i}_{corr}(\mu\:A/c{m}^{2})$	$\:{R}_{p}\left(\varOmega\:c{m}^{2}\right)$	$\:C.Rate(mm/\mathrm{a})$
Blank Steel	− 0.73 ± 0.02	8.77 ± 0.53	2262 ± 136	0.556 ± 0.039
$\:Ni\:coated\:steel\:at\:3\:A/d{m}^{2}$	− 0.40 ± 0.01	5.06 ± 0.30	5527 ± 332	0.032 ± 0.002
Ni-0.5 g/L Ni@SiTiCNO$\:\:at\:3\:A/d{m}^{2}$	− 0.30 ± 0.01	4.28 ± 0.26	18,350 ± 1101	0.0213 ± 0.0015
Ni-0.5 g/L Ni@SiTiCNO$\:\:at\:4\:A/d{m}^{2}$	− 0.20 ± 0.01	1.86 ± 0.11	15,520 ± 931	0.0180 ± 0.0013
Ni-0.5 g/L Ni@SiTiCNO$\:\:at\:5\:A/d{m}^{2}$	− 0.10 ± 0.01	2.43 ± 0.15	12,300 ± 738	0.0220 ± 0.0015
$\:Ni\:coated\:steel\:at\:4\:A/d{m}^{2}$	− 0.30 ± 0.01	3.53 ± 0.21	11,590 ± 695	0.0260 ± 0.0018
Ni-1 g/L Ni@SiTiCNO$\:\:at\:3\:A/d{m}^{2}$	− 0.28 ± 0.01	4.06 ± 0.24	19,431 ± 1166	0.0190 ± 0.0013
Ni-1 g/L Ni@SiTiCNO$\:\:at\:4\:A/d{m}^{2}$	− 0.10 ± 0.01	2.52 ± 0.15	25,220 ± 1513	0.0170 ± 0.0012
Ni-1 g/L Ni@SiTiCNO$\:\:at\:5\:A/d{m}^{2}$	0.08 ± 0.01	1.30 ± 0.08	27,580 ± 1655	0.0160 ± 0.0011
Ni-1.5 g/L Ni@SiTiCNO$\:\:at\:3\:A/d{m}^{2}$	− 0.26 ± 0.01	1.86 ± 0.11	20,520 ± 1231	0.0090 ± 0.0008
Ni-1.5 g/L Ni@SiTiCNO$\:\:at\:4\:A/d{m}^{2}$	− 0.18 ± 0.01	2.61 ± 0.16	32,440 ± 1946	0.0160 ± 0.0011
Ni-1.5 g/L Ni@SiTiCNO$\:\:at\:5\:A/d{m}^{2}$	0.06 ± 0.01	0.92 ± 0.06	3306 ± 198	0.0100 ± 0.0008
$\:Ni\:coated\:steel\:at\:5\:A/d{m}^{2}$	− 0.27 ± 0.01	2.43 ± 0.15	12,300 ± 738	0.0220 ± 0.0015
Ni-2 g/L Ni@SiTiCNO$\:\:at\:3\:A/d{m}^{2}$	− 0.25 ± 0.01	4.27 ± 0.26	23,520 ± 1411	0.0070 ± 0.0008
Ni-2 g/L Ni@SiTiCNO$\:\:at\:4\:A/d{m}^{2}$	− 0.16 ± 0.01	2.69 ± 0.16	30,870 ± 1852	0.0110 ± 0.0008
Ni-2 g/L Ni@SiTiCNO$\:\:at\:5\:A/d{m}^{2}$	− 0.02 ± 0.01	0.84 ± 0.05	40,034 ± 2402	0.0085 ± 0.0008

### Abrasion resistance

**Fig. 17:**
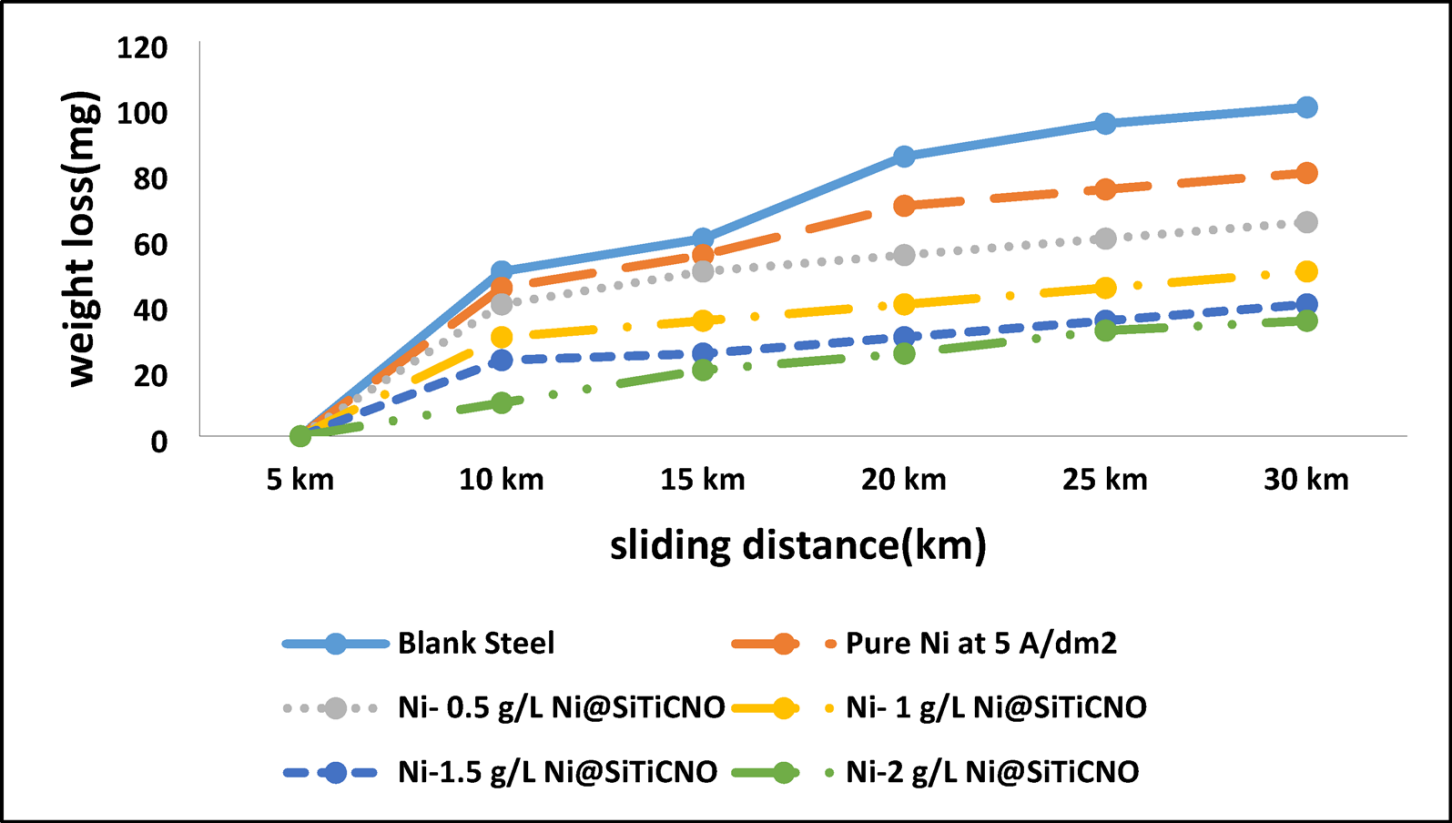
The relation between sliding distance and weight loss of the different Ni@SiTiCNO nano composite coating layers.

A sand-water erosion test was performed with a water-to-sand ratio of 3:2 and a rotational speed of 300 rpm to assess the wear resistance of the unprotected steel, conventional Ni-coated steel, and Ni@SiTiCNO nanocomposite-coated steel. The findings show a clear relationship between sliding distance and material weight loss (Fig. 17). Notably, compared to the traditional Ni coating, Ni@SiTiCNO nanocomposite plating showed noticeably less weight loss, suggesting improved wear resistance. The enhanced hardness of the nanocomposite structure, which successfully resists abrasive forces, may be the cause of this improvement. The data demonstrates how the addition of SiTiCNO nanoparticles improves the mechanical performance and durability of electrodeposited Ni coatings.

In order to improve the mechanical, tribological, and occasionally corrosion-related properties of composite coatings, strengthening mechanisms are essential. The effectiveness of the strengthening mechanisms within the coating structure determines the final performance of composite coatings. The coating’s resistance to corrosion, wear, deformation, and cracking is improved by strengthening mechanisms. Smaller grains in the matrix prevent dislocation movement when reinforcement particles or process conditions cause them to shrink, strengthening and hardening the coating. Grain boundaries prevent plastic deformation and slip^47^.Thus, by limiting dislocation movement, improving wear and overall durability, increasing load transfer between matrix and reinforcements (SiTiCNO nanoparticles), and refining the coating microstructure, strengthening mechanisms in this work significantly improve composite coatings.

## Conclusions

In the current study, Watt’s bath electrodeposition process has been successfully used to create Ni@SiTiCNO composite coatings to improve the surface characteristics of agricultural animal waste trailers. The following are the findings of this study:

- In comparison to pure Ni (10 μm), the optimal operating conditions are 5 A/dm^2^, 250 rpm, and 2 g/L SiTiCNO nanoparticles in the electrolyte to produce Ni@SiTiCNO nanocomposite coating with 12.7 μm thickness and with a high performance.
- The weight% of SiTiCNO in the coating layer increases significantly as the electrolyte’s SiTiCNO concentration rises, reaching its maximum value at 2 g/L.
- The SEM images demonstrated that the perfect Ni crystal shape was achieved by pure Ni and after adding Ni@SiTiCNO the morphology was slightly changed.
- The Ni@SiTiCNO coated surface exhibits higher impedance and a more negative phase angle, indicating effective corrosion protection. The uncoated surface will exhibit lower impedance and a phase angle closer to 0° or slightly negative, indicating the presence of active corrosion. In the Bode plots, at low frequencies, the impedance is highest for the coated samples, showing good resistance to corrosion, while the uncoated steel shows the lowest impedance, indicating poor corrosion resistance.
- The Ni@SiTiCNO nanocomposite coating exhibited the lowest corrosion rate and the highest corrosion resistance, measuring 0.008 mm/a at 5 A/dm^2^ and 2 g/L.
- Finally, the results demonstrate how the addition of SiTiCNO nanoparticles improves the mechanical performance of electrodeposited Ni coatings.

## Supplementary Information

Below is the link to the electronic supplementary material.

Supplementary Material 1

## Data Availability

The data that support the findings of this study are available from the corresponding author.
