# Supplementary material for: The effectiveness of Ni@SiTiCNO nanocomposite coating for protecting steel used in agricultural machinery dealing with animal waste
Source: Sci Rep. 2026 Apr 17;16:12725. doi: 10.1038/s41598-026-47435-4 (PMC13090366; doi:10.1038/s41598-026-47435-4)
Supplement: Supplementary file 1 — Supplementary Material 1 [file 41598_2026_47435_MOESM1_ESM.docx]

# Table S1: displayed the SiC-Si₃N₄-TiC-TiO₂/SiO₂ (SiTiCNO) nanocomposite synthesis conditions.

Table S1: Synthesis Preparation Method for SiTiCNO

| Step | Process Description | Conditions / Notes |
| --- | --- | --- |
| 1 | Molar Ratio | Ti:Si:C = 1:1:8 |
| 2 | Silica sol-gel/ pH | 3.0 |
| 3 | Hydrolysis of titanium sol | At 70°C for 2 hours |
| 4 | Combine binary sols | Closed Teflon beaker for 3 days |
| 5 | Dry mixed gel | at 110°C |
| 6 | Carbothermal Reduction Reaction | Heat to 600°C at 5°C/min, hold 1 h under argon Heat to 1500°C under Ar/N₂ (~300 cm³/min)  Hold at 1500°C for 3 hours |

The binary Ti-Si gel was formulated with a molar ratio of Ti:Si:C = 1:1:8. Silicon and titanium sols were prepared separately. For the silicon sol: lactose (C₁₂H₂₂O₁₁) was dissolved in 50 milliliters of DIW (deionized water) at 70 degrees Celsius and cooled. The lactose slurry was then supplemented with tetraethyl orthosilicate (TEOS), which had been previously combined with ethanol. After stirring to homogeneity, the solution's pH (which was initially 3.7) was brought down to 3.0 by adding 0.01 M nitric acid dropwise.

Titanium tetraisopropoxide (TTIP) is gradually added to the agitated isopropanol solution to create the titanium sol-gel. The obtained mixture was stirred for 15 min, and furthermore, deionized water was added drop-wise to this mixture. A white precipitate was appeared then it converted to white solution. The resulted solution was kept under stirring at 70 degrees Celsius for 2 h. Appreciate amount of acetic and nitric acid was added in sequence with slowly added. The final resulting solution was allowed to stir for 2 h.

After that, the binary sols were raised collectively in a closed Teflon® beaker at room temperature for three days to fully create the mixed gel network. To get rid of extra water and solvent, the mixed gel was dried at 110 degrees Celsius. The xerogel gel was weighed after it had aged and dried. In the middle of a tube furnace (Carbolite Furnace Limited), the xerogel was positioned inside an alumina tube. The furnace was fixed at 600 degrees Celsius for one hour in an argon flow after being heated from room temperature at a rate of 5 degrees Celsius per minute. Then, in an argon/nitrogen flow, the temperature was raised at the same rate to 1500 degrees Celsius; the flow rate was roughly 300 cm³/min. To aid in the carbothermal reduction, the temperature was maintained at 1500 degrees Celsius for three hours. SiTiCNO is the nominated sample.


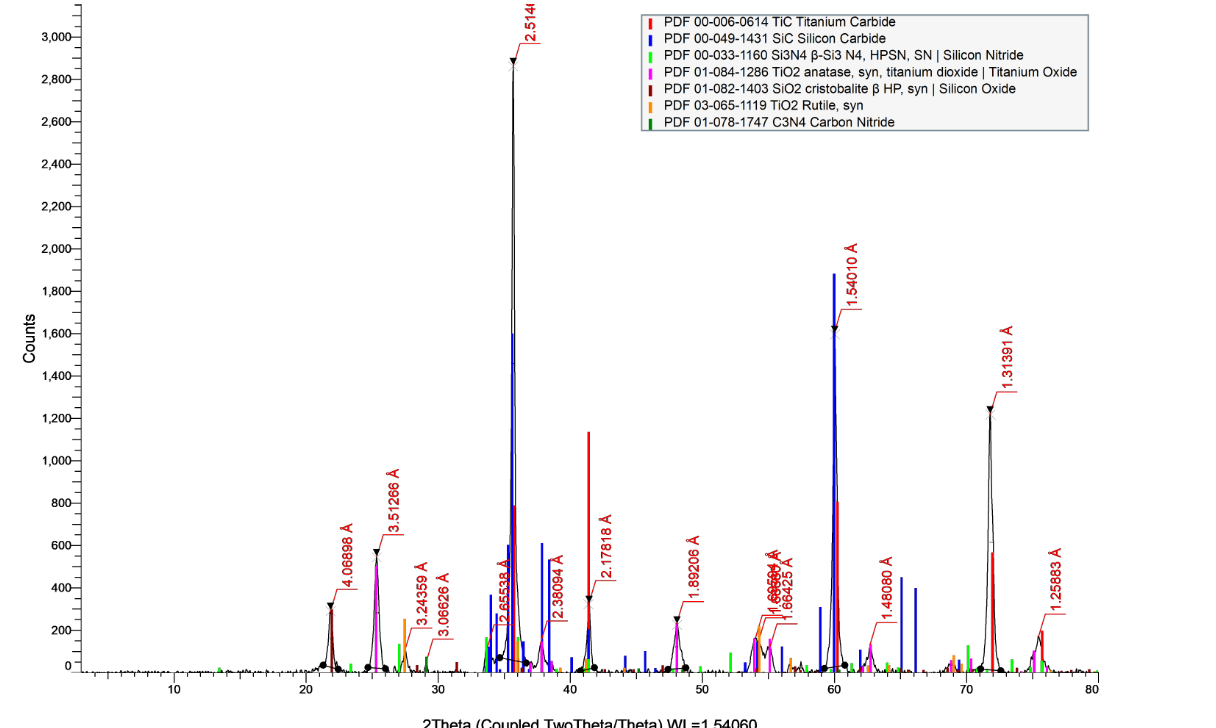


b)

**2θ**

**Fig. S1. The X-ray diffraction (XRD) chart illustrates patterns of the synthesized powder.**

***
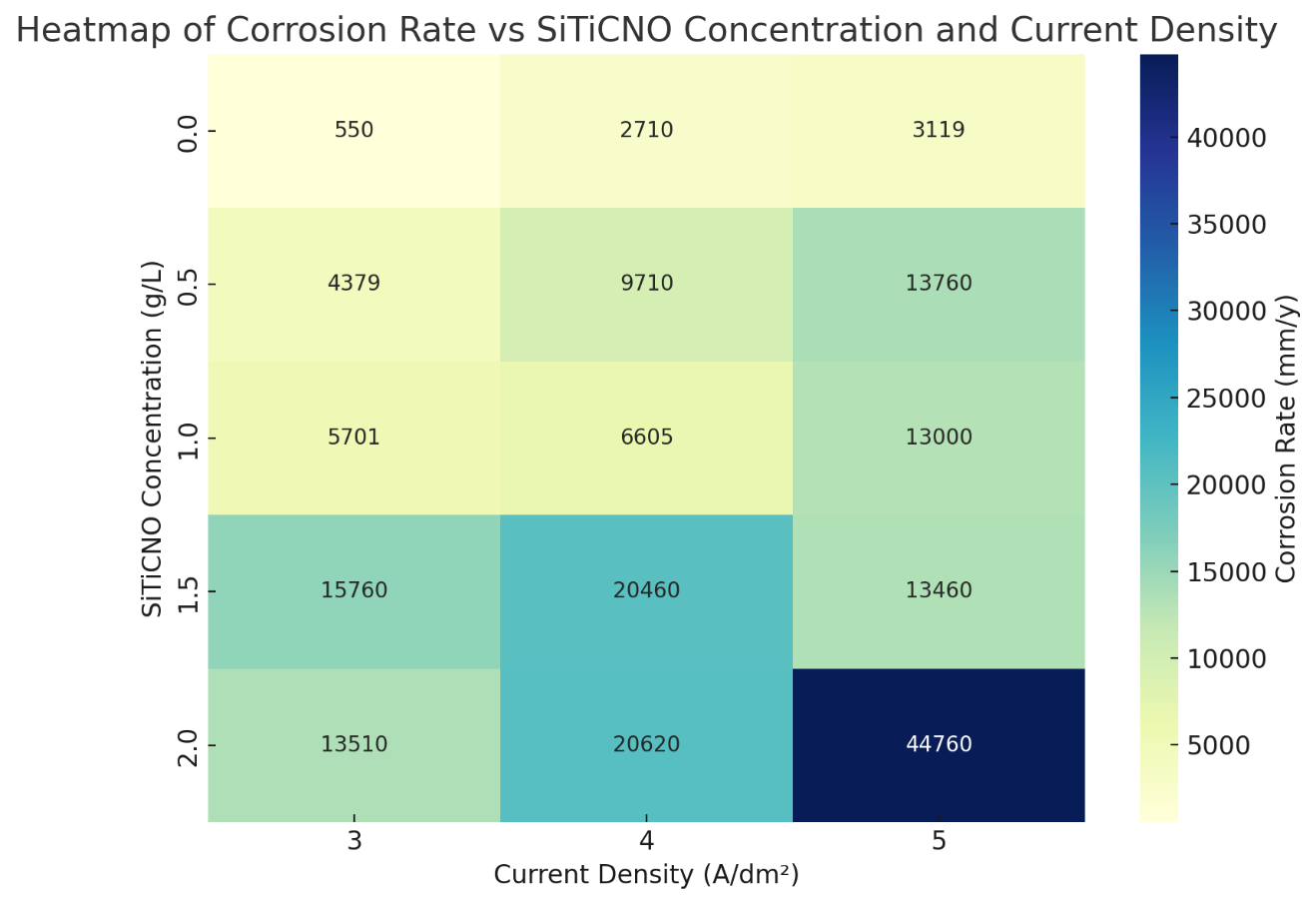
***

**Fig. S2. Heatmap for uncoated steel, Ni-coated steel and Ni@SiTiCNO nanocomposite-coated steel using different concentrations of Ni@SiTiCNO**
